# Distinct phylogenetic relationships and biochemical properties of Arabidopsis ovarian tumor-related deubiquitinases support their functional differentiation

**DOI:** 10.3389/fpls.2014.00084

**Published:** 2014-03-12

**Authors:** Ramalingam Radjacommare, Raju Usharani, Chih-Horng Kuo, Hongyong Fu

**Affiliations:** Institute of Plant and Microbial Biology, Academia SinicaTaipei, Republic of China

**Keywords:** Arabidopsis, deubiquitylation, OTU, ubiquitin, DUB

## Abstract

The reverse reaction of ubiquitylation is catalyzed by different classes of deubiquitylation enzymes (DUBs), including ovarian tumor domain (OTU)-containing DUBs; experiments using *Homo sapiens* proteins have demonstrated that OTU DUBs modulate various cellular processes. With the exception of OTLD1, plant OTU DUBs have not been characterized. We identified 12 *Arabidopsis thaliana OTU* loci and analyzed 11 of the encoded proteins *in vitro* to determine their preferences for the ubiquitin (UB) chains of M1, K48, and K63 linkages as well as the UB-/RUB-/SUMO-GST fusions. The *A. thaliana* OTU DUBs were shown to be cysteine proteases and classified into four groups with distinct linkage preferences: OTU1 (M1 = K48 > K63), OTU3/4/7/10 (K63 > K48 > M1), OTU2/9 (K48 = K63), and OTU5/11/12/OTLD1 (inactive). Five active OTU DUBs (OTU3/4/7/9/10) also cleaved RUB fusion. OTU1/3/4 cleaved M1 UB chains, suggesting a possible role for M1 chains in plant cellular signaling. The different substrate specificities of the various *A. thaliana* OTU DUBs indicate the involvement of distinct structural elements; for example, the OTU1 oxyanion residue D89 is essential for cleaving isopeptide bond-linked chains but dispensable for M1 chains. UB-binding activities were detected only for OTU2 and OTLD1, with distinct linkage preferences. These differences in biochemical properties support the involvement of *A. thaliana* OTU DUBs in different functions. Moreover, based on the established phylogenetic tree, plant- and *H. sapiens*-specific clades exist, which suggests that the proteins within these clades have taxa-specific functions. We also detected five OTU clades that are conserved across species, which suggests that the orthologs in different species within each clade are involved in conserved cellular processes, such as ERAD and DNA damage responses. However, different linkage preferences have been detected among potential cross-species OTU orthologs, indicating functional and mechanistic differentiation.

## Introduction

By modulating the stability, activity, interaction, or subcellular localization of critical regulatory and mechanistic components, the covalent attachment and removal of ubiquitin (UB), i.e., ubiquitylation and deubiquitylation, are essential mechanistic and regulatory elements of numerous cellular processes, such as chromatin silencing, transcriptional activation, mRNA splicing and export, cell division, DNA damage response, intracellular trafficking, and signal transduction (Hershko and Ciechanover, 1998; Komander et al., 2009; Reyes-Turcu et al., 2009).

The close connection between reversible ubiquitylation and almost all aspects of cellular processes and organismic functions is primarily due to the many components involved, which include substrates, conjugation enzymes, deconjugation enzymes, and diverse signals derived from the small but highly conserved UB proteins and their cognate binding partners, which decipher the signals (Smalle and Vierstra, 2004; Fu et al., 2010). The numerous protein-interacting interfaces of UB and attachment as a monomer or polymers with distinct linkages enables UB assembly on substrates to yield diverse signals (Komander and Rape, 2012). The attachment of monomeric UB and UB chains through eight different linkages, including the linear M1, K6, K11, K27, K29, K33, K48, and K63 linkages, produces different topoisomers with distinct functional roles. While K48-linked UB chains target modified proteins for proteasomal degradation (Chau et al., 1989), K63-linked chains are critical for signaling complex assembly, endocytosis, and DNA damage responses (Chen and Sun, 2009). K11- and K29-linked chains are alternative proteasomal degradation signals for cell-cycle regulators and UB fusion degradation pathway substrates, respectively (Johnson et al., 1995; Jin et al., 2008). More recently, linear UB chains and typical K63 linkages have emerged as important signals for NF-κB activation pathways in mammals (Rieser et al., 2013). The cellular functions of the other atypical chains remain elusive (Chen and Sun, 2009; Kulathu and Komander, 2012).

While conjugation enzymes, including activation enzymes (E1s) and, in particular, the UB carriers (E2s) and ligases (E3s), are essential for UB signal assembly on substrates, the reverse reaction, which is catalyzed by a diverse set of deubiquitylation enzymes (DUBs), is equally important for modulating the formation and disassembly of the diverse ubiquitin signals. DUBs have been extensively studied in budding yeast (*Saccharomyces cerevisiae*) and mammals for their roles in various cellular and organismic processes and their biochemical properties, including structural elements, catalytic mechanisms, UB binding, and substrate specificities (Reyes-Turcu et al., 2009). Similar to those from other eukaryotic species, Arabidopsis (*Arabidopsis thaliana*) DUBs are grouped into five classes and comprise the second most abundant enzymatic component of the UB system (Komander et al., 2009). Four of these classes are cysteine proteases, including 3 UB C-terminal hydrolases (UCH) (Yang et al., 2007), 27 UB-specific processing proteases (UBP/USP) (Yan et al., 2000; Liu et al., 2008), 12 *ovarian tumor*(OTU)-related proteases (this study), and 2 Josephin/Machado-Joseph disease proteases (MJD) (At3g54130 and At2g29640). The other class, belonging to the zinc-dependent metalloproteases, comprises at least 6 JAB1/MPN/Mov34 (JAMN) domain-containing proteases, including AMSH1-3, the proteasome subunit RPN11, and BRCC36A/B (Yang et al., 2004; Isono et al., 2010; Block-Schmidt et al., 2011).

Based on phenotype analyses of T-DNA insertion mutants, *A. thaliana* DUBs of various classes play important roles in plant growth and development. However, the specific cellular processes and mechanistic components involved have not been determined for most of the plant DUBs described. Among the UCH DUBs, UCH1 and UCH2 are likely involved in auxin signaling and are critical for shoot architecture and leaf morphology (Yang et al., 2007). The best characterized plant DUBs are the UBPs, including UBP1 and UBP2, which are required for the turnover of aberrant proteins (Yan et al., 2000); UBP3 and UBP4, which are essential for male gametophyte development (Doelling et al., 2007); UBP12 and UBP13, which play as negative regulators in immune response (Ewan et al., 2011); UBP14 and UBP19, which are important for embryogenesis (Doelling et al., 2001; Liu et al., 2008); and UBP15, which is critical for vegetative and reproductive growth (Liu et al., 2008). UBP26 is involved in both transcriptional suppression and activation. It is required for seed development through modulation of the repressive histone mark H3K27me3 on the Polycomb group complex-targeted gene *PHERES1* (Luo et al., 2008). UBP26 is also required for heterochromatic silencing of transgenes and transposons by affecting the methylation of DNA and histone H3 at various lysines (Sridhar et al., 2007). By contrast, UBP26 is required for transcriptional activation of *FLOWERING LOCUS C (FLC)* to suppress flowering (Schmitz et al., 2009). In the *ubp26* mutant, ubH2B accumulates globally and at the *FLC* locus. Moreover, the activating histone mark H3K36me3 and the repressive mark H3K27me3 were decreased and increased, respectively, at the *FLC* locus, resulting in transcriptional suppression. Among the JAMN DUBs, AMSH1 and AMSH3 are likely involved in deubiquitylating endocytosed plasma membrane cargos by interacting with the ESCRT-III subunits (Katsiarimpa et al., 2011, 2013). BRCC36A and BRCC36B are homologs of mammalian BRCC36, a component of a protein complex containing BRCA1, and are likely involved in intra- and inter-chromosomal homologous recombination (Block-Schmidt et al., 2011). With the exception of OTLD1, the functional and biochemical properties of the *A. thaliana* OTU DUBs have not been characterized. OTLD1 may function in a histone-modifying repressor complex harboring the histone lysine demethylase KDM1C to suppress specific gene expression through histone deubiquitylation and demethylation (Krichevsky et al., 2011).

The OTU domain was initially identified in the *Drosophila melanogaster OTU* gene product (Steinhauer et al., 1989) and subsequently observed in protein sequences from various eukaryotes, including animals and plants, viruses, and a single bacterium *Chlamydia pneumonia* (Makarova et al., 2000). The *Homo sapiens* OTU domain-containing proteins OTUB1 and OTUB2 were subsequently identified as novel DUBs in experiments employing UB derivatives with thiol-reactive C-terminal groups (Borodovsky et al., 2002). Subsequent extensive biochemical, structural, and functional analyses have primarily been conducted with *H. sapiens* OTU DUBs; these studies have provided extensive information on their biochemical properties and important regulatory roles in signaling cascades.

The phylogenetic relationships between OTU DUBs from different species have not been reported. A phylogenetic tree established for *H. sapiens* OTU proteins identified four major clades: the OTUB clade (OTUB1 and OTUB2), the OTUD clade (OTUD1, OTUD2/YOD1, OTUD3, OTUD4, OTUD5/DUBA, OTUD6A, OTUD6B, and ALG13), the A20-like clade (A20/TNFAIP3, Cezanne/OTUD7B, Cezanne2/OTUD7A, TRABID/ZRANB1, and VCPIP1/VCIP135), and the OTULIN clade (OTULIN) (Mevissen et al., 2013). Members of the A20 and OTULIN clades are the most extensively studied *H. sapiens* OTU DUBs. A20, OTUD7B, and OTULIN generally play a negative regulatory role in various NFκB signaling pathways (Boone et al., 2004; Wertz et al., 2004; Enesa et al., 2008; Hitotsumatsu et al., 2008; Turer et al., 2008; Hymowitz and Wertz, 2010; Fiil et al., 2013; Hu et al., 2013; Keusekotten et al., 2013; Rivkin et al., 2013). The OTUB clade members, particularly OTUB1, have also been extensively characterized. OTUB1 is involved in immune responses (Soares et al., 2004), estrogen receptor-mediated transcription (Stanišiæ et al., 2009), and the DNA damage response (Nakada et al., 2010). The other, less-examined *H. sapiens* OTU DUBs and their potential orthologs from other species also play important roles in various cellular processes and signaling pathways. OTUD5/DUBA regulates interferon signaling (Kayagaki et al., 2007). Whereas VCIP1 is involved in CDC48-mediated Golgi membrane fusion (Wang et al., 2004), *H. sapiens* YOD1 and its possible yeast ortholog Otu1 are involved in p97/CDC48-mediated ERAD (Rumpf and Jentsch, 2006; Ernst et al., 2009).

In contrast to the characterized USPs, which have promiscuous linkage preferences (Faesen et al., 2011), the *H. sapiens* OTU DUBs have more strict linkage specificities (Mevissen et al., 2013). The distinct linkage specificities associated with OTU DUBs could be exploited in restriction analyses to determine the linkage types of the ubiquitin chains conjugated on endogenous substrates (Fiil et al., 2013; Hospenthal et al., 2013; Mevissen et al., 2013), to purify ubiquitylated substrates with specific linkage types, and to assemble UB chains with specific linkage(s) (Bremm et al., 2010). Such restriction applications are essential tools for ubiquitylation biology. Extensive biochemical and structural analyses have revealed multiple mechanisms for determining the linkage specificities of OTU DUBs, including the presence of additional UB binding domain(s), ubiquitylation sites, S1' and S2 substrate sites on the OTU core, and substrate-assisted catalysis (Wang et al., 2009; Juang et al., 2012; Licchesi et al., 2012; Wiener et al., 2012; Keusekotten et al., 2013; Mevissen et al., 2013). However, to precisely manipulate the linkage specificities of particular OTU DUBs, more extensive biochemical and structural analyses are required to determine the exact molecular bases underlying the selectivity for UB dimers and UB polymers of specific linkages.

Given the potential importance of *A. thaliana* OTU DUBs in plant growth and development, we identified 12 *A. thaliana* loci encoding OTU DUBs. We characterized the biochemical properties (i.e., substrate binding and specificities) of 11 *A. thaliana OTU* loci-encoded proteins with a complete OTU domain. The *A. thaliana* OTU DUBs were classified into four groups with distinct substrate binding and specificities. Based on the phylogenetic tree established using OTU sequences from different species, conserved and plant-specific OTU DUBs were identified. Distinct substrate specificities were observed among possible cross-species orthologs within the same phylogenetic clades, suggesting potential mechanistic and functional differentiation. The distinct biochemical properties and phylogenetic relationships of *A. thaliana* OTU DUBs support their functional differences. Based on the phylogenetic tree, we discuss the possible unique and conserved functional roles of *A. thaliana* OTU DUBs.

## Results

### *A. thaliana* has an OTU-DUB family with 12 phylogenetically distinct members

To characterize *A. thaliana* OTU-DUBs, 12 loci, *OTU1-5, OTLD1, and OTU7-12*, encoding OTU domain-containing proteins were identified through database searches using the OTU domain sequences for *H. sapiens* OTUB1 (NP_060140) and OTUB2 (NP_075601) as well as *S. cerevisia*e Otu1 (P43558) as the initial queries (Table 1). The exon-intron organization and coding sequences of these *A. thaliana* loci were determined through a sequence comparison with the corresponding PCR-amplified full-length cDNAs as well as available cDNAs and ESTs from the TAIR database (http://www.arabidopsis.org/) (Figure S1). Based on the splicing products detected, a single isoform exists for the *OTU1-3*, *OTU9-10*, and *OTU12* loci; two isoforms exist for the *OTU5*, *OTLD1*, *OTU7*, and *OTU11* loci (referenced with *a* and *b* extensions); and five isoforms exist for *OTU4* (*OTU4a-e*) (Table 1; Figure S1). With the exception of *OTU4b* and *OTU4d-e*, for which the encoded proteins were predicted to have lost or disrupted OTU domains due to frame shifts from alternative splicing, the remaining *OTU* loci encode potential OTU domain-containing DUBs. However, *OTU8* is likely a pseudogene because corresponding multiple isolated cDNAs were consistently derived from alternative splicing using a different 3′ junction of the second annotated intron (61 bp upstream of the predicted junction), which generated a frame-shift and downstream start codon located on the third annotated exon, resulting in an N-terminally truncated OTU domain. Accordingly, a search of the GENEVESTIGATOR microarray databases (https://www.genevestigator.com/gv/plant.jsp) revealed extremely low expression levels for *OTU8* transcripts and moderate to high expression levels for *OTU1-2*, *OTU4-5, OTLD1*, *OTU9*, and *OTU11-12* transcripts across various primary and cultured tissues from different organ sources (Figure S2). Microarray probes are not available for *OTU3*, *OTU7*, and *OTU10*. In general, constitutive expression of the eleven active *OTU* loci in various tissues, including roots, inflorescence stems, rosette and cauline leaves, flowers, and siliques, was detected by RT-PCR (Figure S3).

**Table 1:** ***A. thaliana* loci encoding OTU-DUBs**.

**Gene**	**Locus**	**Peptide length (aa)/MW (kDa)**	**Domains identified (coordinates)^a^**	**Full-length cDNA accession^b^**
*OTU1*	At2g28120	306/34.4	Peptidase_C65 (41–294)	JQ013442
			OTU (87–289)	
*OTU2*	At1g50670	208/23.4	OTU (11–123)	JQ013443
*OTU3*	At2g38025	234/26.3	OTU (82–230)	JQ013444
*OTU4a*	At3g57810	317/35.8	OTU (174–300)	JQ013445
*OTU4b*		217/24.2	OTU^*^ (174–216)	BX824976
*OTU4c*		274/30.8	OTU (131–257)	JQ013446
*OTU4d*		195/21.8	OTU^*^ (131–191)	JQ013447
*OTU4e*		75/8.3	ND	JQ013448
*OTU5a*	At3g62940	332/37.4	OTU (187–321)	JQ013449
			Coiled coil (19–86)	
*OTU5b*		316/35.7	OTU (172–306)	JQ013450
			Coiled coil (4–71)	
*OTLD1a*	At2g27350	506/55.3	OTU (222–333)	JQ013451
			UBA-like (453–491)	
*OTLD1b*		505/55.3	OTU (222–333)	AY058065
			UBA-like (452–490)	
*OTU7a*	At5g67170	375/41.5	OTU (43–155)	JQ013452
			SEC-C (313–333)	
*OTU7b*		231/26.9	OTU (43–155)	JQ013453
*OTU8*	At2g39320	154/18.3	OTU^*^ (1–62)	JQ013454
			Coiled coil (85–150)	
*OTU9*	At5g04250	345/39.2	OTU (210–322)	JQ013455
*OTU10*	At5g03330	356/41.7	OTU (219–331)	JQ013456
*OTU11a*	At3g22260	245/28.2	OTU (107–219)	JQ013457
*OTU11b*		240/27.7	OTU (107–219)	JQ013458
*OTU12*	At3g02070	219/25.6	OTU (85–197)	JQ013459

a The proteins encoded by OTU4b, OTU4d, OTU4e, and OTU8 had disrupted (OTU^*^) or lost (ND) OTU domains due to frame shifts caused by alternative splicing.

b With the exception of OTU4b and OTLD1b, for which full-length cDNAs are available in the TAIR database, the full-length cDNAs for the various His- or His/GST-tagged wild-type recombinant constructs were isolated from various cDNA libraries by PCR (Table S1).

**Figure 1:**
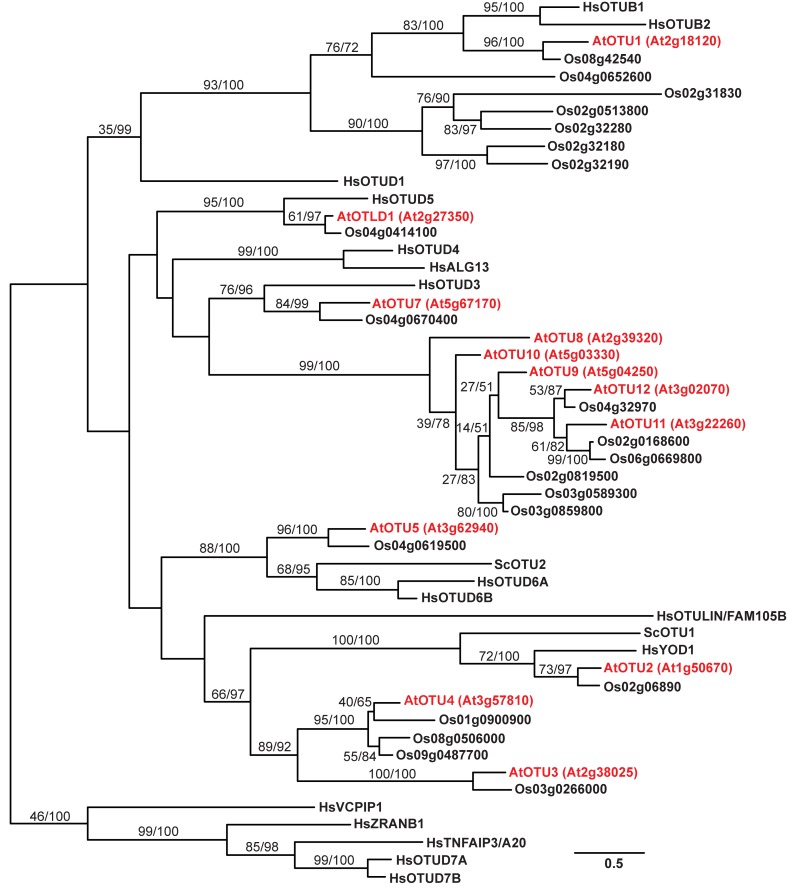
**A phylogenetic tree for the OTU proteins from *A. thaliana*, *O. sativa*, *S. cerevisiae*, and *H. sapiens*.** The phylogenetic tree was generated using aligned OTU domain sequences from *A. thaliana*, *O. sativa*, *H. sapiens*, and *S. cerevisiae*. The branch labels separated by a slash indicate bootstrap support and Bayesian posterior probability. The branch length is based on the maximum likelihood results, and the scale represents the branch length that is proportional to the number of amino acid substitutions. The sequences from *A. thaliana*, *O. sativa*, *H. sapiens*, and *S. cerevisiae* are identified by the prefixes At, Os, Hs, and Sc, respectively, as abbreviations of their binomial names. The *A. thaliana* OTUs are marked in red with corresponding locus numbers in parentheses. The accession numbers for the analyzed sequences are listed in the Materials and Methods.

To examine the phylogenetic relationships between the 12 potential *A. thaliana* OTU-DUBs, a phylogenetic tree (Figure 1) was generated based on the aligned peptide sequences from the OTU domains derived from the *A. thaliana*, *Oryza sativa*, *H. sapiens*, and *S. cerevisiae* OTU proteins. With the exception of OTU8-12, which are in the same clade, each of the remaining *A. thaliana* OTU proteins belongs to a distinct clade in the predicted phylogenetic tree. A single potential *O. sativa* ortholog is associated with each of the *A. thaliana* OTU2-3, OTU5, OTLD1, and OTU7 clades. However, multiple potential *O. sativa* orthologs are associated with the clades containing *A. thaliana* OTU1, OTU4, and OTU9. The closer phylogenetic relationship among the *A. thaliana* and *O. sativa* OTU orthologs within the same clades compared with OTU proteins from the same species in different clades indicates that the orthologs within a clade evolved before the divergence of dicot and monocot species. Among the *A. thaliana* and *O. sativa* orthologs in the same clade, the nearly identical exon/intron organization of their corresponding loci further supports their close phylogenetic relationship (Figure S1). Five clades harbor OTU proteins derived from *A. thaliana*, *O. sativa*, and *H. sapiens*; the two clades containing *A. thaliana* OTU2 and OTU5 also include *S. cerevisiae* Otu1 and Otu2, respectively. The five clades conserved across species likely evolved before their common ancestors diverged. *A. thaliana* OTU1, OTU2, OTU5, OTLD1, and OTU7 belong to the same clades as *H. sapiens* OTUB1/OTUB2, *H. sapiens* YOD1/*S. cerevisiae* Otu1, *H. sapiens* OTUD6A/OTUD6B/*S. cerevisiae* Otu2, *H. sapiens* OTUD5, and *H. sapiens* OTUD3 respectively, which suggests that these *A. thaliana* OTU proteins have distinct, evolutionarily conserved functions. No plant orthologs are associated with the four *H. sapien*s-specific clades, which include OTUD1; OTUD4/ALG13; the arbitrary outgroup clade harboring VCPIP1, ZRANB1, TFNAIP3/A20, OTUD7A, and OTUD7B; and the OTULIN/FAM105B clade. Similarly, three plant-specific clades harboring *A. thaliana* OTU3, OTU4, and OTU8-12 with one or multiple *O. sativa* homologs are present.

### *A. thaliana* OTU-DUBs exhibit distinct substrate specificities

The lengths of the encoded *A. thaliana* OTU proteins range from 208 to 506 amino acids, with predicted molecular weights of ~23.4–55.3 kDa (Table 1). Each protein includes an OTU domain (PFAM accession number: PF02338) with a length of 113–203 amino acids. Similar to its *H. sapiens* counterparts OTUB1 and OTUB2, *A. thaliana* OTU1 includes a larger Peptidase_C65 domain (PF10275) that covers the OTU domain. The OTU domain is located at the center of OTLD1; the C-terminal region of OTU1, OTU3-5, and OTU9-12; and the N-terminal region of OTU2 and OTU7. All of the *A. thaliana* OTU domains detected include the conserved catalytic Cys-His-Asp/Asn triad, similar to known active OTU-DUBs such as *H. sapiens* OTUB1 (Figure S4). In addition to the OTU domain, a few known domains were identified that are associated with the *A. thaliana* OTU proteins, including a coiled-coil domain in OTU5a (19–86) and OTU5b (4–71), a SCOP UBA-like domain (d1fya) in OTLD1a (453–491) and OTLD1b (452–490), and a potential nucleic acid-binding SEC-C motif (PF02810) in OTU7a (313–333) (Table 1).

The distinct phylogenetic relationships of the *A. thaliana* OTU proteins strongly suggest functional differentiation. To further explore this possibility, we characterized the differences in their biochemical properties, including deubiquitylation substrate specificities and binding activities against K48- and K63-linked UB chains. Therefore, we purified recombinant wild-type, site-specific, and deletion variants of the *A. thaliana* OTU proteins, which were expressed in *Escherichia coli* as His-tagged or His- and glutathione *S*-transferase (His/GST)-double tagged proteins (Table S1). The deubiquitylation substrate specificities of the *A. thaliana* OTU proteins OTU1-3, OTU4c-d, OTU5b, OTLD1a, OTU7a, OTU9-10, OTU11a, and OTU12 were examined (Table 2). With the exception of OTU4c and OTU10, which were examined using the His/GST-tagged versions due to low yields of their His-tagged forms, the His-tagged versions were analyzed. His/GST-tagged OTU1 was also analyzed and compared with the His-tagged form to examine whether fusion of the large GST tag affected catalytic activity. We expected that the His-tagged OTU4d would be catalytically defective due to a truncated OTU domain. His-tagged *H. sapiens* OTUB1 was analyzed as a control and for comparison. Other OTU isoforms were not analyzed for various reasons, such as low yields of both tagged recombinant proteins (OTU4a), small sequence variation from the analyzed isoforms (OTU5a, OTLD1b and OTU11b), a large C-terminal truncation (OTU7b), and lost or disrupted OTU domains (OTU4b, OTU4e, and OTU8).

**Table 2:** **Distinct biochemical properties of *A. thaliana* OTU-containing deubiquitinases**.

**Name^a^**	**Tag**	**Ub4**		**Linear chain**			**Ub/Ubl-GST fusion**			**Chain^b^**
		**K48**	**K63**	**Ub2**	**Ub3**	**Ub4**	**Ub**	**Rub**	**Sumo**	
OTU1	His	+++^c^	+	+++	+++	+++	−	−	−	−
OTU1	GST	+++	+	+++	+++	+++	−	−	−	**−**
OTU2	His	+	+	−	−	−	−	−	−	K48 ≈ K63
OTU3	His	++	+++	−	+	+	++	+	−	−
OTU4c	GST	+++	+++	−	++	++	++	+	−	−
OTU4d	His	−	−	−	−	−	−	−	−	−
OTU5b	His	−	−	−	−	−	−	−	−	−
OTLD1a	His	−	−	−	−	−	−	−	−	K48 > K63
OTU7a	His	+	++	−	−	−	++	+	−	−
OTU9	His	++	++	−	−	−	++	+	−	−
OTU10	GST	++	+++	−	−	−	++	+	−	−
OTU11a	His	−	−	−	−	−	−	−	−	−
OTU12	His	−	−	−	−	−	−	−	−	−
HsOTUB1	His	+++	−	−	−	−	−	−	−	ND

a OTU4d contains a disrupted OTU domain.

b UB chain binding was analyzed by GST pull-down assays using GST-fused proteins; –, absence of binding; ND, not determined.

c The approximate deubiquitylation activities are quantitatively designated by +++, ++, +, and − for strong, moderate, weak, and absent activity, respectively.

**Figure 2:**
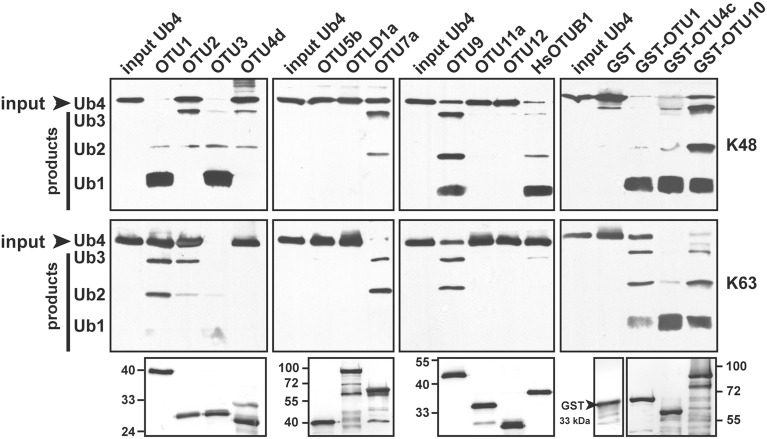
**The *A. thaliana* OTU-containing proteins display distinct cleavage preferences for K48- and K63-linked UB tetramers.** *A. thaliana* OTU proteins cleavage activities for K48- and K63-linked UB tetramers (Ub4) were analyzed using purified His- (OTU1-3, OTU4d, OTU5b, OTLD1a, OTU7a, OTU9, OTU11a, and OTU12) or His/GST-tagged (OTU1, OTU4c, and OTU10) forms. The His-tagged *H. sapiens* otubain 1 (HsOTUB1) was analyzed for comparison. GST was included as a negative control for His/GST-tagged OTU proteins. A substrate incubated without enzymes was used as a negative input control (input Ub4). The inputs and their cleavage products are labeled on the left as trimers (Ub3), dimers (Ub2), and monomers (Ub1) and were visualized by immunoblotting using antisera against *H. sapien*s UB (α-UB). Duplicate input OTU proteins were visualized through immunoblotting using antisera against the His-tag to ensure approximately equivalent enzyme input levels (bottom panels). The molecular weight markers are labeled on the left or right.

The substrate specificities of the potential *A. thaliana* OTU DUBs were examined by determining their *in vitro* cleavage activities on isopeptide bond-linked UB tetramers with K48- and K63-linkages; peptide bond-linked linear UB dimers, trimers, and tetramers; and peptide bond-linked UB-, RUB-, and SUMO-GST fusion proteins. As summarized in Table 2, seven of the 11 examined *A. thaliana* OTU proteins exhibited deubiquitylation activities with distinct substrate specificities. The selectivity of the potential *A. thaliana* OTU DUBs for K48- and K63-linked UB chains was first determined (Table 2; Figure 2). Both His- and His/GST-tagged OTU1, similar to its *H. sapiens* counterpart OTUB1, displayed a clear preference for K48-linked UB chains. However, unlike OTUB1, which did not exhibit detectable activity for K63-linked chains, *A. thaliana* OTU1 displayed moderate activity with this chain linkage. By contrast, while OTU4c displayed a slight cleavage preference for K63-linked UB chains, OTU3, OTU7a, and OTU10 displayed stronger cleavage activities for K63-linked UB chains. However, OTU2 and OTU9 exhibited approximately similar cleavage activities for both linkage types. The remaining *A. thaliana* OTU proteins examined, OTU5b, OTLD1a, OTU11a, and OTU12, did not exhibit detectable *in vitro* cleavage activity for either chain linkage or other tested substrates, which suggests these proteins are cryptic *in vitro* or have specificity for other, unexamined chain linkages. As predicted, the OTU4d with a disrupted OTU domain displayed no significant cleavage activity against either chain linkage.

**Figure 3:**
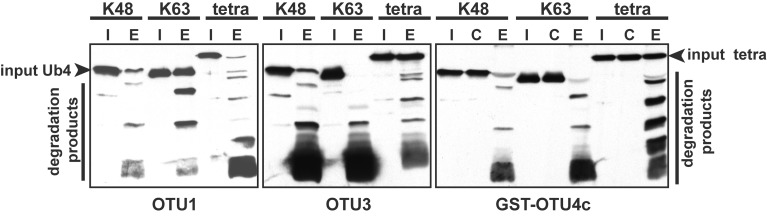
**The relative cleavage activities of *A. thaliana* OTU1, OTU3, and OTU4c against linear (tetra) and K48- or K63-linked UB tetramers.** The cleavage activities were analyzed using His-tagged OTU1 and OTU3 and His/GST-tagged OTU4c (E). GST was included as a negative control for GST-OTU4c (C). A substrate incubated without enzymes was used as a negative input control (I). The inputs and their cleavage products are labeled on the left or right; the degradation products were visualized by immunoblotting with α-UB.

**Figure 4:**
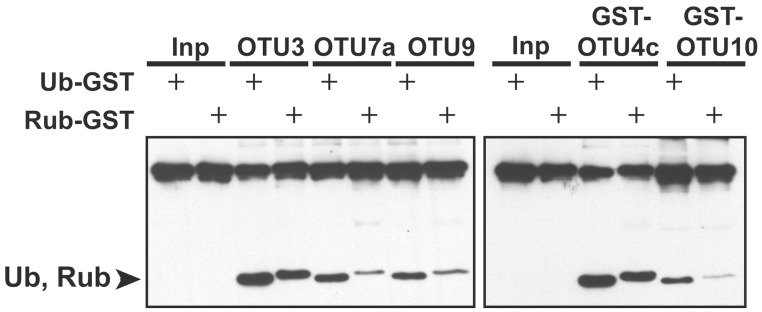
**The relative cleavage activities of *A. thaliana* OTU3, OTU4c, OTU7a, OTU9, and OTU10 for UB- and RUB-fused GST.** The cleavage activities were analyzed using His-tagged OTU3, OTU7a, and OTU9 as well as His/GST-tagged OTU4c and OTU10. The substrates incubated without enzymes were used as negative input controls (Inp). The specific substrate used is indicated by “+.” The cleavage products Ub and Rub, which are labeled on the left, and the inputs were visualized by immunoblotting with α-HA.

Among the seven *A. thaliana* OTU proteins active against isopeptide bond-linked UB chains, OTU1, OTU3, and OTU4c also exhibited cleavage activities against peptide bond-linked linear UB polymers. Both His- and His/GST-tagged OTU1 proteins exhibited strong cleavage activities with all tested linear UB polymers, and OTU3 and OTU4 exhibited weak to moderate cleavage activities with linear UB trimers and tetramers but not dimers (Table 2; Figure 3; Figure S5). Moreover, whereas *A. thaliana* OTU1 exhibited similarly stronger catalytic activities for peptide bond- and K48 isopeptide bond-linked UB tetramers than for K63-linked UB tetramers, OTU3 and OTU4c had stronger catalytic activities for isopeptide bond-linked UB tetramers of both linkages than peptide bond-linked linear UB tetramers (Figure 3). Interestingly, in contrast to the strong catalytic activities detected with *A. thaliana* OTU1, the *H. sapiens* OTUB1 was inactive with peptide bond-linked linear UB chains (Table 2; Figure S5). With the exception of OTU1 and OTU2, the active *A. thaliana* OTU proteins also displayed cleavage activities against peptide bond-linked UB- and RUB-GST fusion proteins but not the SUMO-GST fusion protein (Table 2; Figure 4; Figure S6). A cleavage preference for UB-GST over RUB-GST was evident (Table 2; Figure 4).

We examined the cleavage activities for isopeptide-bond linked ubiquitin tetramers with K48 and K63 linkages; OTU1, OTU3, OTU4c, OTU7a, OTU9, and OTU10 generally displayed optimum activity at neutral pH (Figure S7). However, for K63-linked chain cleavage, OTU9 and OTU10 exhibited optimum activity at slightly alkaline pH values. These neutral and slightly alkaline pH optima suggest that the active *A. thaliana* OTU DUBs examined are likely not vacuolar proteases.

### *A. thaliana* OTU-DUBs are cysteine proteases with a conserved catalytic triad

**Figure 5:**
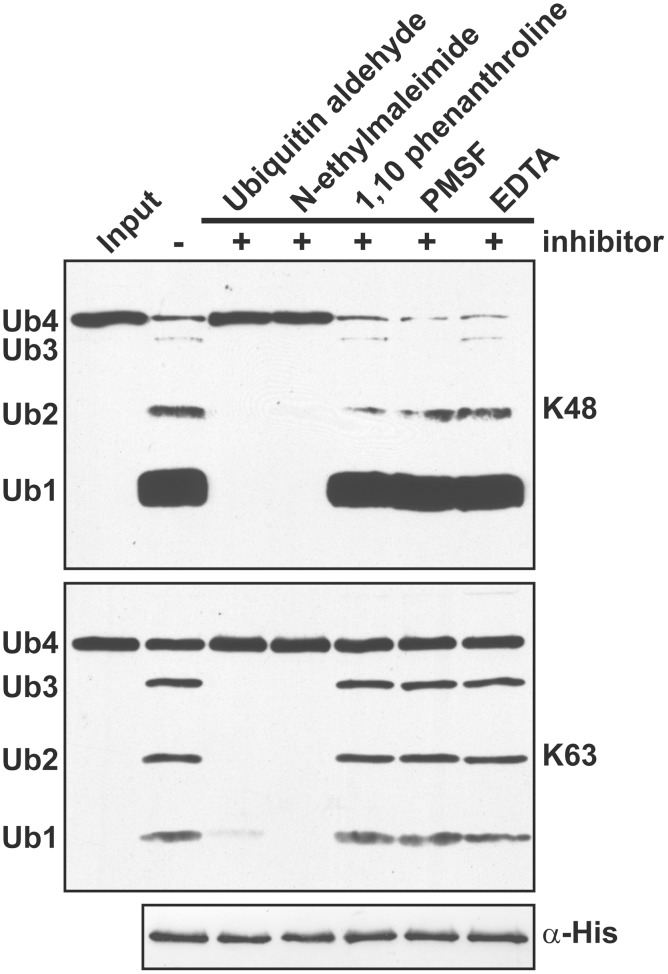
***A. thaliana* OTU1 is catalytically inhibited by cysteine protease inhibitors.** The effects of different protease inhibitors on the cleavage activity of His-tagged *A. thaliana* OTU1 (300 nM) for K48- and K63-linked UB tetramers (250 ng) were examined by comparing the resulting cleavage products after pre-incubation (5 min at 4°C) and the catalytic reaction (1 h at 37°C) in the absence (−) and presence (+) of 0.5 μM UB aldehyde, 0.5 μM N-ethylmaleimide, 0.5 μM 1,10-phenanthroline, 1 mM phenylmethylsulfonyl fluoride (PMSF), or 1 mM EDTA. The input substrate incubated without OTU1 was used as a negative control (Input). The input Ub4 and cleavage products Ub3, Ub2, and Ub1, labeled on the left, were detected by immunoblotting with α-UB. Duplicate samples were examined using antisera against the His-tag to confirm that OTU1 was input at equivalent levels (α-His).

**Figure 6:**
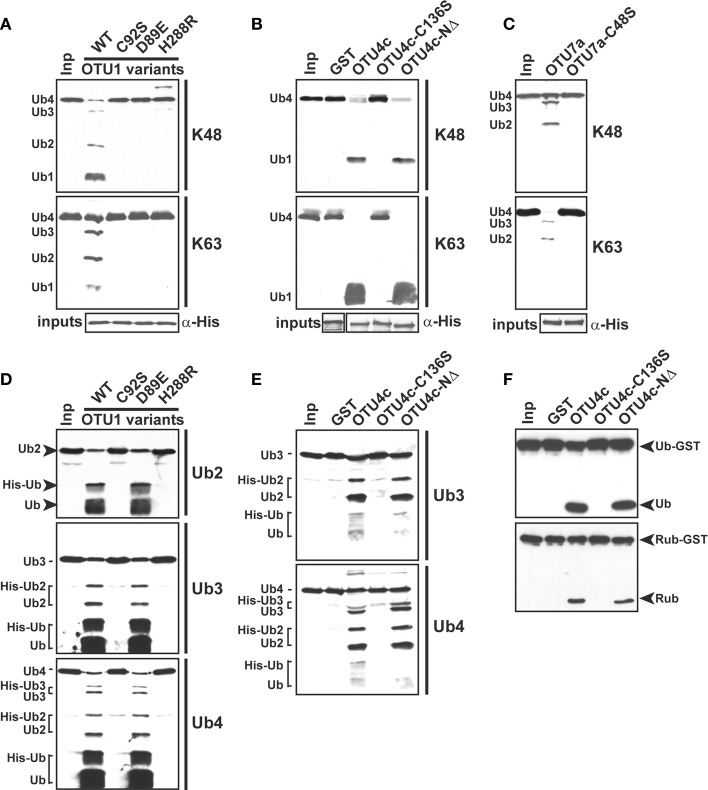
**The conserved catalytic triad is required for the deubiquitylation activities of *A. thaliana* OTU1, OTU4c, and OTU7a. (A)–(E)** The catalytic roles of the conserved D89 in OTU1, the conserved catalytic triad in OTU1, OTU4c, and OTU7a, and the 43 N-terminal residues in OTU4c for cleavage of K48- and K63-linked UB tetramers **(A)–(C)**, peptide bond-linked linear UB dimers (Ub2), trimers (Ub3), and tetramers (Ub4) **(D,E)**, and peptide bond-linked UB- and RUB-GST fusions **(F)** were examined by comparing the cleavage activities of the site-specific or deletion mutants, which included the OTU1 variants C92S, D89E, and H288R; OTU4c-C136S; OTU4c-NΔ; and OTU7a-C48S, with those of the corresponding wild-type proteins. Substrates incubated without enzyme were used as negative input controls (Inp). The inputs and their cleavage products are labeled on the left or right and were visualized by immunoblotting with α-UB **(A)–(E)** or α-HA **(F)**. Duplicate input OTU variants were detected by immunoblotting with antisera made against His-tag to confirm equivalent enzyme input levels (α-His). The His- (OTU1 and OTU7a variants) or His/GST-tagged (OTU4c variants) forms were used. GST was included as a negative control for the GST-fused OTU4c variants.

The presence of a conserved catalytic triad (Figure S4) suggests that the *A. thaliana* OTU DUBs are cysteine proteases similar to those characterized from *H. sapiens* and *S. cerevisiae*. As tested using the His-tagged OTU1, the cleavage activities for K48- and K63-linked UB tetramers were inhibited by UB aldehyde and N-ethylmaleimide but not by the metalloprotease inhibitors 1,10-phenanthroline and EDTA or the serine protease inhibitor phenylmethylsulfonyl fluoride (Figure 5). Furthermore, site-specific mutations of the conserved catalytic triad in OTU1, OTU4c, and OTU7a or of a conserved residue that is potentially critical for stabilizing the oxyanion reaction intermediate in OTU1 (D89) completely abolished cleavage activities for K48- and K63-linked UB tetramers (Figures 6A–C; OTU1 variants C92S, D89E, and H288R, OTU4c-C136S, and OTU7a-C48S). The same mutations also generally abolished the linear UB polymer cleavage activities of OTU1 and OTU4c (Figures 6D,E) and the UB- and RUB-GST fusion protein cleavage activities of OTU4c (Figure 6F). Interestingly, although cleavage of isopeptide bond-linked UB tetramers was abolished, OTU1-D89E still cleaved linear UB polymers to a similar extent as the wild-type protein (Figure 6D).

*A. thaliana* OTU1 D89 and corresponding Asp residues in other OTU DUBs, including *H. sapiens* OTUB1 and OTUB2, were initially predicted to be involved in catalytic triad formation by polarizing active-site histidine (Balakirev et al., 2003). Instead, crystal structure analyses revealed that either an Asp or Asn (D290 of OTU1) located one residue away in the C-terminal direction from the active-site histidine is involved in catalytic triad formation through hydrogen bonds to orient the active-site histidine (Nanao et al., 2004; Edelmann et al., 2009). However, the backbone amides of the originally predicted Asp residues of OTUB1, OTUB2, A20, and yeast Otu1 (corresponding to OTU1 D89) are critical for stabilizing the oxyanion reaction intermediate (Nanao et al., 2004; Komander and Barford, 2008; Edelmann et al., 2009).

The substrate selectivity of various *A. thaliana* OTU DUBs may be an intrinsic property of their respective OTU domains. An N-terminal 43-residue deletion in OTU4c did not modify its catalytic activities with the examined isopeptide and peptide bond-linked substrates (Figures 6B,E,F; OTU4c-NΔ). Similarly, the cleavage of K48- and K63-linked tetra ubiquitin chains by N-terminal deletion variants of OTU9 and OTU10 (Table S1, OTU9-NΔ1-100, OTU9-NΔ1-172, OTU10-NΔ1-125, and OTU10-NΔ1-173) containing intact OTU domains was comparable to that catalyzed by the corresponding wild-type proteins (data not shown).

### *A. thaliana* OTU2 and OTLD1 bind UB chains with distinct linkage preferences

DUBs including OTUs often harbor UB-binding domains (Komander et al., 2009). An appendage of the UB-binding domain(s) may restrict or broaden the linkage specificities, as has been observed for UIM, ZnF, and an ankyrin repeat domain in *H. sapiens* OTUD1, OTUD2, and TRABID, respectively (Licchesi et al., 2012; Mevissen et al., 2013). Additional UB-binding domain(s), such as the multiple NZFs of TRABID, are involved in targeting to substrate sites and enhance cleavage activity with longer UB chains (Licchesi et al., 2012). Furthermore, the different C-terminal linkage-specific UB chain-binding motifs (ZnF1-7) in *H. sapiens* A20 are crucial for its recruitment by distinct signaling proteins and complexes (Bosanac et al., 2010; Skaug et al., 2011; Tokunaga et al., 2012; Verhelst et al., 2012; Lu et al., 2013).

**Figure 7:**
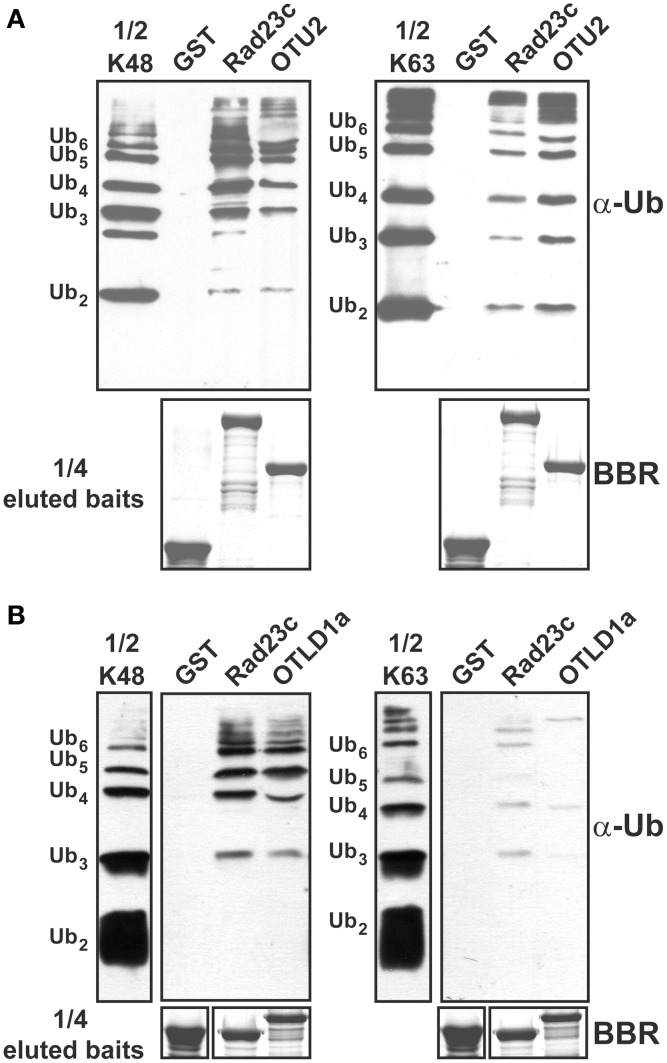
**OTU2 and OTLD1 associate with isopeptide bond-linked UB chains and have distinct preferences for different linkage types. (A)** OTU2 exhibited equivalent binding affinities for K48- and K63-linked UB chains. **(B)** OTLD1a prefers to bind K48- over K63-linked UB chains. Five micrograms of K48- or K63-linked UB chains (2–6 units, labeled on the left) were subjected to pull down by GST-fused OTU2 **(A)**, OTLD1a **(B)**, and negative (GST) and positive (AtRAD23c) control proteins. One microgram of the input chains (1/2 K48 or K63) and two-fifths of the eluted pulled-down products were analyzed by immunoblotting with α-Ub. One-tenth of the eluted pulled-down products (1/4 eluted baits) were visualized by Brilliant Blue R staining (BBR) to confirm equally immobilized baits.

Because binding preferences for UB chains with different linkages may contribute to substrate selectivity or intracellular targeting, we examined the association between the *A. thaliana* OTU proteins and K48- and K63-linked UB chains *in vitro* through pull-down assays using the GST-fusion proteins (Table S1). OTU1-3, OTU4c, OTU5b, OTLD1a, OTU7a, OTU9-10, OTU11a, and OTU12 were examined. However, binding of K48- and K63-linked UB chains was detected only for OTU2 and OTLD1a (Table 2; Figures 7A,B). OTU2 exhibited approximately equivalent binding affinities for K48- and K63-linked UB chains, while OTLD1a displayed a clear binding preference for K48-linked UB chains.

### OTU2 and OTLD1 harbor novel UB-binding domains

**Figure 8:**
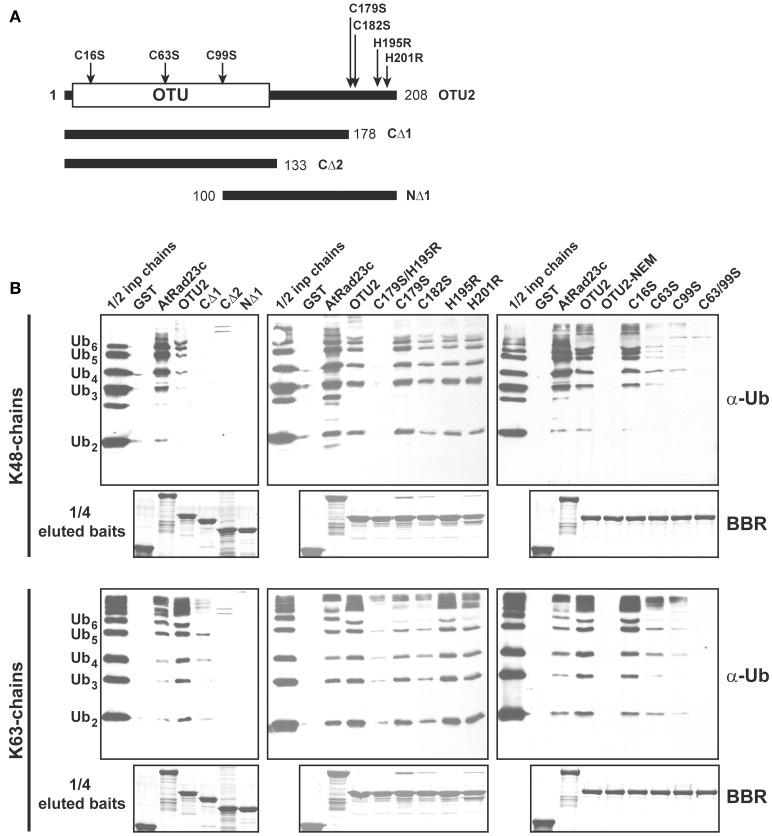
**Residues located at both the OTU2 N- and C-termini are critical for ubiquitin chain binding. (A)** Schematic diagrams of the site-specific and deletion variants of OTU2 that were used to delineate the domains and residues involved in UB binding. The site-specific mutations and OTU domain are indicated. The numbers indicate the coordinates for the OTU2 variant termini. **(B)** GST pull-down products. Five micrograms of K48- or K63-linked UB chains (2–6 units, labeled on the left) were subjected to pull down by GST-fused OTU2 variants and negative and positive control proteins (GST and AtRAD23c, respectively). One microgram of the input chains (1/2 inp chains) and two-fifths of the eluted pulled-down products were analyzed by immunoblotting using antisera against human UB (α-Ub). One-tenth of the eluted pulled-down products (1/4 eluted baits) were visualized by Brilliant Blue R staining (BBR) to confirm equivalently immobilized baits. Wild-type OTU2 pre-incubated with 0.5 μM *N*-ethylmaleimide was analyzed to examine the involvement of catalytic and non-catalytic cysteine residues (OTU2-NEM).

**Figure 9:**
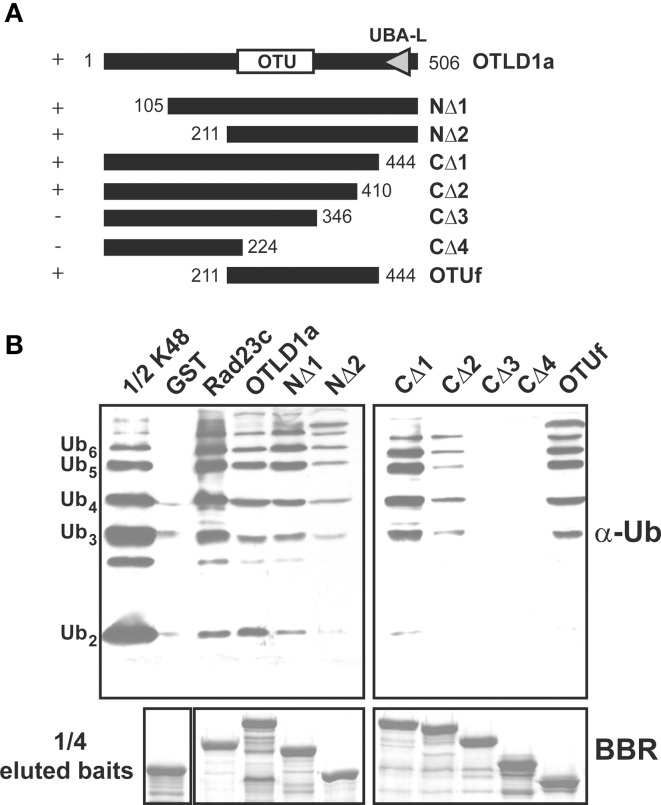
**The OTLD1a region spanning 211–410 but not the UBA domain is critical for UB chain binding. (A)** Schematic diagrams show the OTLD1a deletion variants that were used to delineate the domain involved in UB chain binding. The OTU and UBA-like (UBA-L) domains are indicated. Positive and negative binding activity against K48-linked UB chains are indicated by + and − on the left. The numbers indicate the OTLD1a variant termini coordinates. **(B)** Pull-down products of the GST-fused OTLD1a variants. Five micrograms of K48-linked UB chains (2–6 units, labeled on the left) were subject to pull down by GST-fused OTLD1a variants and negative and positive control proteins (GST and AtRAD23c, respectively). One microgram of the input chains (1/2 K48) and two-fifths of the eluted pulled-down products were analyzed by immunoblotting with α-Ub. One-tenth of the eluted pulled-down products (1/4 eluted baits) were visualized by Brilliant Blue R staining (BBR) to confirm equivalently immobilized baits.

We delineated the critical domains or residues involved using GST-fused deletion and/or site-specific variants (Figures 8A, 9A; Table S1). Binding assays using OTU2 terminal truncations indicated that both the N- (1–99) and C-terminal regions (179–208) contain structural elements that are critical for binding K48- and K63-linked UB chains. The association with K48- and K63-linked UB chains was abolished for all OTU2 terminal deletion mutants (Figures 8A,B; CΔ1, CΔ2, and NΔ1). A PROSITE Zinc_Finger_C2H2 motif (http://prosite.expasy.org/PS00028) containing four well-spaced Cys and His residues was identified at the OTU2 C-terminus (179–201). Individual mutants of the Cys and His residues in this domain still bound to the UB chains with affinities nearly equivalent to that of the wild-type protein (Figures 8A,B; C179S, C182S, H195R, and H201R). However, the double mutant C179S/H195R exhibited completely abolished (K48-linked chain) or severely affected (K63-linked chain) binding. These results also support a critical role for the OTU2 C-terminus in UB-chain association. The predicted zinc finger motif likely does not exist because any single mutation of the predicted residues involved in zinc chelation would have disrupted the zinc finger and C-terminal structure. Because the OTU2 N-terminal region containing the OTU domain is critical for chain binding, OTU2 was treated with NEM to determine if the catalytic cysteine is essential. Association with both UB chain types was abolished when OTU2 was pre-incubated with NEM, suggesting a critical role for the catalytic cysteine in chain binding (Figure 8B; OTU2-NEM). However, the catalytic cysteine (C16) is actually not essential for UB chain binding because the site-specific mutant bound UB chains as well as the wild-type protein (Figure 8B; C16S). The deleterious effect of NEM on OTU2 UB chain binding indicates that other Cys residues are likely critical. We examined the roles of two other available Cys residues (C63 and C99) in the N-terminal half of OTU2 in UB chain association. Association with both chain types was almost completely abolished in OTU2 variants with single and double substitutions of these Cys residues (Figures 8A,B; C63S, C99S, and C63S/99S). Most known UB-binding domains and motifs, such as UIM and UBA, are formed from small and restricted UB-binding protein regions, and thus the involvement of scattered critical residues/domains in UB chain association by OTU2 is unique and suggests that a novel structural domain formed from a broader sequence region is likely involved in UB binding.

Although OTLD1a contains a UBA-like domain at the C-terminus, a C-terminal-truncated variant in which the UBA-like domain was removed exhibited K48-linked chain binding activity equivalent to that of the wild-type protein (Figures 9A,B; CΔ1). Based on additional experiments with various terminal deletion variants, the UB chain binding region is likely positioned between residues 211–410; N-terminal deletion variants truncated before residue 211 and C-terminal deletion variants truncated after 410 bound K48-linked chains (Figures 9A,B; NΔ1-2 and CΔ1-4). An internal fragment (211–444) encompassing OTU domain (OTUf) also bound K48-linked chains.

## Discussion

We characterized *A. thaliana* OTU domain-containing DUBs; based on extensive studies from other species, these proteins likely function as critical mechanistic elements with important cellular and organismic processes. The distinct phylogenetic relationships and biochemical properties observed suggest that the *A. thaliana* OTU-DUBs identified are likely involved in different functions.

Similar to OTU DUBs characterized from other species, the *A. thaliana* OTU DUBs are cysteine proteases; as determined with OTU1, OTU4, and OTU7 by the importance of the conserved catalytic triad and an oxyanion residue and through specific inactivation with cysteine protease inhibitors. Based on *in vitro* DUB assays, the *A. thaliana* OTU DUBs have distinct substrate preferences. Because ubiquitin dimers with all eight linkages have become available more recently (El Oualid et al., 2010; Virdee et al., 2010; Hospenthal et al., 2013) and most long atypical chains, such as K6, K27, K33, and K29 are not available, we analyzed only the linear (M1-linked), K48-linked, and K63-linked UB chains. Seven of the 11 *A. thaliana* OTU domain-containing proteins exhibited deubiquitylation activities with distinct substrate preferences. We categorize the *A. thaliana* OTU DUBs into four groups based on substrate preferences (Table 2; Figures 2–4). The first group consists of a single member, OTU1, which exhibited equivalently stronger preferences for linear and K48-linked UB chains than that for K63-linked chains. The second group includes OTU3, OTU4, OTU7, and OTU10, which exhibited slightly or clearly stronger preferences for K63-linked chains than K48-linked and linear chains. OTU3 and OTU4 had weak to moderate cleavage activities for linear UB tetramers and trimers, and OTU7 and OTU10 were inactive toward the linear UB chains. The third group contains OTU2 and OTU9, which exhibited nearly equivalent preferences for K48- and K63-linked UB chains and no cleavage activity toward linear UB chains. The fourth cryptic group includes OTU5, OTLD1, OTU11, and OTU12, which were inactive against all substrates examined *in vitro*.

The absence of *in vitro* cleavage activity for OTU5, OTLD1, OTU11, and OTU12 did not completely rule out their potential function as DUBs *in vivo* because only a limited number of substrates were tested. A cryptic or auto-inhibited conformation has been associated with DUBs of various classes, including OTUs, that have been examined *in vitro* (Nanao et al., 2004; Messick et al., 2008). Moreover, catalytic activation of a specific cryptic enzyme may require association with additional factor(s) or assembly into protein complex(es). DUBs of various classes, including OTUs, often function in complexes (Sowa et al., 2009). The cryptic enzyme can also be activated by post-translational modifications such as phosphorylation, as has been observed for *H. sapiens* OTUD5 (the possible *A. thaliana* OTLD1 ortholog), for which phosphorylation is critically involved in substrate binding (Huang et al., 2012). The conserved OTU5 catalytic cysteine is irreplaceable for rescuing mutant phenotypes, which supports the notion that its deubiquitylation activity is functionally relevant and that it is active *in vivo* (Radjacommare and Fu, unpublished results). Thus, it is worth examining the cryptic OTU DUBs for their *in vitro* cleavage activities toward ubiquitin chains with other linkages that have recently become available and for their importance of the catalytic sites *in vivo*. It is also worth identifying the interacting proteins/substrates for the cryptic group of *A. thaliana* OTU proteins. However, some of the inactive *A. thaliana* OTU DUBs may be involved in non-catalytic functions, as demonstrated for OTUB1 and A20 (Nakada et al., 2010; Skaug et al., 2011; Juang et al., 2012).

Although we examined cleavage activities with only three UB linkages, we compared the linkage preferences of the *A. thaliana* OTU DUBs with potential orthologs in other species, particularly *H. sapiens*. Differences on linkage preference have been clearly detected among the potential orthologs from different species, suggesting functional and/or mechanistic differentiation. A strict linkage preference is generally associated with OTU DUBs in *H. sapiens*-specific clades as determined using UB dimers of all eight linkages (Mevissen et al., 2013). OTUD1, OTUD4, OTUD7A/OTUD7B, and OTULIN have strict preferences for K63-, K48-, K11-, and M1-linked UB chains, respectively. However, the other three *H. sapiens*-specific OTUs have cryptic (ALG13), dual- (A20 and VCPIP1, K11/K48), or multiple (TRABID/ZRANB1, K29 = K33 > K63) linkage preferences. Among OTU DUBs in the five conserved phylogenetic clades, whereas both *H. sapiens* and *Caenorhabditis elegans* OTUB1 orthologs have strict specificity for K48 linkages (Wang et al., 2009; Mevissen et al., 2013), *A. thaliana* OTU1 has equally strong preferences for linear and K48-linked chains over K63-linked chains. The other *H. sapiens* OTUB1 homolog, OTUB2, also has distinct substrate preferences; it cleaves K63-linked chains with greater activity than K48- and K11-linked chains. In addition, although the orthologs from *H. sapiens* (YOD1/OTUD2), *S. cerevisiae* (Otu1), and *C. elegans* (Otu1) have generally similar multiple cleavage activities (K29 = K33 = K27 > K11 > K48), *A. thaliana* OTU2 has equivalently moderate activities for K48- and K63-linked UB chains. Moreover, whereas the potential *H. sapiens* ortholog (OTUD3) has dual cleavage preferences for K6- and K11-linked chains, *A. thaliana* OTU7 has greater cleavage activity for K63-linked chains than K48-linked chains. By contrast, two potential orthologs, *A. thalian*a OTU5 and *H. sapiens* OTUD6B, from the same phylogenetic clade are both inactive for each linkage examined. However, a slightly more divergent *H. sapiens* ortholog, OTUD6A, has cleavage activity for multiple linkages (K29 = K33 = K27 > K11 > K63) (Mevissen et al., 2013). Because *H. sapiens* OTUD5/DUBA is activated by phosphorylation with dual preferences (K6 = K11) (Huang et al., 2012; Mevissen et al., 2013), potential activation through phosphorylation and the linkage specificity of the potential *A. thaliana* ortholog OTLD1, which is inactive in our DUB assay, must be further investigated. However, for an extensive cross-species comparison of linkage specificities among OTU DUBs, more extensive *in vitro* assays with UB dimers or longer UB chains with all linkages are necessary.

With the exception of OTU1, which has equivalent peptidase and isopeptidase activities and exhibits similarly strong cleavage activities for linear and K48-linked ubiquitin chains, the active *A. thaliana* OTU DUBs are generally isopeptidases. However, although OTU3 and OTU4 have stronger preferences for K63 isopeptide bond-linked chains, both OTU DUBs also have weak to moderate activities for linear ubiquitin chains. In contrast to *A. thaliana*, among 16 *H. sapiens* OTU DUBs with a complete catalytic triad, only the recently discovered OTULIN has strict cleavage activity for linear M1-linked UB chains. Modulation of linear ubiquitin chain assembly and disassembly has emerged as an important regulatory mechanism for signal transduction in mammals (Iwai, 2011; Rieser et al., 2013). The observed cleavage activities for linear chains by *A. thaliana* OTU DUBs, particularly OTU1, suggest a potential functional relevance of linear UB chains in plants. However, linear chain assembly components of the E3 ligase LUBAC are restricted to vertebrates and select invertebrate lineages and are not found in plants (Keusekotten et al., 2013).

In contrast to human OTU DUBs, which are all inactive against NEDD8 (RUB)-based peptide substrates (Edelmann et al., 2009; Mevissen et al., 2013), five of the seven active *A. thaliana* OTU DUBs (OTU3-4, OTU7, OTU9, and OTU10 but not OTU1 and OTU2) have cleavage activities for UB- and RUB-GST fusion proteins, with a clear preference for UB-GST. Although RUB-GST is an artificial substrate, the activities detected for this substrate indicate that *A. thaliana* OTU DUBs may play a role in modulating RUB conjugation, which is important for regulating CULLIN-based E3 ligase activities (Stratmann and Gusmaroli, 2012).

The distinct substrate preferences of various *A. thaliana* OTU DUBs suggest structural differences in the substrate recognition mechanisms. The strict preferences of OTU1 and OTU2 for peptide- and/or isopeptide bond-linked UB chains but not UB- and RUB-GST fusion proteins suggest that contacts with both distal and proximal UBs are critical for cleavage. Similarly, *H. sapiens* OTUB1 does not cleave UB-GFP (Balakirev et al., 2003). The importance of proximal UB binding has been demonstrated with *H. sapiens* OTULIN, which is inactive against active-site probes such as UB propargylamide and UB-AMC (Keusekotten et al., 2013; Rivkin et al., 2013). The remaining *A. thaliana* OTU DUBs can cleave UB- and RUB-GST fusion proteins, which suggests that the leaving group (either UB or GST) has little influence on catalysis. However, the clear linkage preferences among these OTU DUBs still favor the structural differences surrounding the isopeptide linkage at the proximal UB (S1′) binding sites. The importance of S1′ binding sites for linkage specificity has been established for *H. sapiens* OTUB1, OTULIN, and OTUD1-3 (Wang et al., 2009; Juang et al., 2012; Wiener et al., 2012; Keusekotten et al., 2013; Mevissen et al., 2013). Using *H. sapiens* OTUD1-3, conserved S1′ binding sites have been observed between orthologs from different species, but these binding sites diverged significantly between different OTUD families (Mevissen et al., 2013).

It is generally believed that linear and K63-linked UB chains have similar structural conformations (Trempe, 2011; Komander and Rape, 2012). However, *A. thaliana* OTU1 can discriminate between these linkages, and it selectively cleaves linear and K48-linked chains over K63-linked chains, indicating structural differences between linear and K63-linked UB chains. The presence of structural differences between linear and K63-linked chains is also supported by the different cleavage activities of OTU3-4, OTU7, and OTU10 toward these linkages. The structural elements that are critical for OTU1 recognition are likely similar in linear and K48-linked UB chains but diverge in K63-linked chains. Alternatively, *A. thaliana* OTU1 may utilize distinct recognition elements in the cleavage of linear and K48-linked UB chains. Consistent with the latter notion, the critical OTU1 residues involved in catalyzing isopeptide bond-linked or peptide bond-linked UB chains are not identical; the Asp residue (D89) is critical for cleaving isopeptide bond-linked chains but is dispensable for cleaving linear chains (Figure 6), which indicates a role for D89 in stabilizing an oxyanion reaction intermediate that is relevant when cleaving isopeptide bond-linked chains but not linear chains. Different OTU1 residue(s) are likely used to stabilize the oxyanion reaction intermediate for linear chain cleavage.

Among the eleven *A. thaliana* OTU DUBs examined, we found that OTU2 and OTLD1 bind UB chains with distinct linkage preferences. Although a ZnF-like motif and UBA-like domain were identified in OTU2 and OTLD1, respectively, the ZnF-like structure likely does not exist in OTU2, and OTLD1 UBA is dispensable for UB-binding, suggesting the involvement of novel domains. Consistent with our results, neither a bound zinc ion nor the ZnF domain was detected in the yeast ortholog Otu1 in crystal structural analyses (Messick et al., 2008). In addition, UB binding to the predicted ZnF-like domain region of the potential *H. sapiens* ortholog YOD1/OTUD1 was not detected in nuclear magnetic resonance experiments (Mevissen et al., 2013). Interestingly, both the N- and C-terminal domains/residues of OTU2 are critical for binding to UB chains, which suggests that the UB-binding structure is formed from a broad region of the protein. However, the functions of the UB binding activities in *A. thaliana* OTU2 and OTLD1 must be further investigated.

Although the UB binding assays were conducted at conditions (4°C and absence of DTT) unfavorable for UB cleavage, the detection of UB chain binding activities could be compromised with OTU DUBs (OTU1, OTU3, OTU4, OTU7, OTU9, and OTU10) that are active with the tested linkage types; because the tested K48- and K63-linked chains could be cleaved during the prolonged pull-down assays. It is thus necessary to further examine UB chain binding preferences for those active OTU DUBs using inactive variants and additional linkage types. Moreover, the functional relevance of any detected UB binding activities needs to be further examined.

Based on the phylogenetic tree established in this study, both *H. sapiens*- and plant-specific clades exist, which suggests that these OTU DUBs are involved in taxa-specific functions. The three plant-specific clades include the clade harboring *A. thaliana* OTU8-12, which also harbors six *O. sativa* homologs; the clade harboring *A. thaliana* OTU3/*O. sativa* Os03g0266000; and the clade harboring *A. thaliana* OTU4/*O. sativa* Os01g0900900/Os08g0506000/Os09g0487700. The functions of the plant-specific OTU DUBs are unknown. Notably, a large plant-specific clade harbors *A. thaliana* OTU8-12 and 6 *O. sativa* orthologs, suggesting a functional importance of this clade. The *H. sapiens*-specific clades include OTUD1, OTUD4/ALG13, OTULIN (FAM105B), and VCPIP1/ZRANB1 (TRABID)/TNFAIP3 (A20)/OTUD7A/OTUD7B; the latter two clades generally have important regulatory functions in NFκB signaling (Lee et al., 2000; Evans et al., 2001, 2003; Wertz et al., 2004; Keusekotten et al., 2013). The human-specific VCPIP1 is involved in fundamental p97-mediated reassembly of mitotic organelles (Uchiyama et al., 2002; Wang et al., 2004), which suggests that alternative plant DUB(s) in plant-specific OTU or another class are likely involved in similar processes.

Five clades conserved across species harbor potential orthologs from *A. thaliana*, *O. sativa*, and *H. sapiens*. Two of these conserved clades also harbor potential orthologs from *S. cerevisiae*. Specifically, *A. thaliana* OTU1, OTU2, OTU5, OTLD1, and OTU7 as well as their corresponding *O. sativa* orthologs belong to the clades harboring *H. sapiens* OTUB1/OTUB2, *H. sapiens* YOD1/*S. cerevisiae* Otu1, *H. sapiens* OTUD6A/OTUD6B/*S. cerevisiae* Otu2, *H. sapiens* OTUD5 (DUBA), and *H. sapiens* OTUD3, respectively. Consistent with the relationships in the phylogenetic tree, nearly identical gene structures were observed among the *A. thaliana* and *O. sativa* orthologs. Moreover, the potential *A. thaliana*, *H. sapiens*, and *S. cerevisiae* orthologs from the conserved clades have similar domain organization and high sequence identities/similarities over extended regions (Figure S8).

The potential cross-species orthologs in each of the five cross-species conserved OTU DUB clades are likely devoted to similar cellular functions. Because *S. cerevisiae* Otu1 and *H. sapiens* YOD1 are involved in ERAD (Rumpf and Jentsch, 2006; Ernst et al., 2009), the potential *A. thaliana* ortholog OTU2 may also be involved in ERAD. However, we observed distinct linkage specificities between *A. thaliana* OTU2 and *H. sapiens*, *S. cerevisiae*, and *C. elegans* orthologs, as described above. In addition, whereas both *H. sapiens* YOD1/OTUD2 and *S. cerevisiae* Otu1 interact with p97/Cdc48 through an N-terminal UBL or UBX domain, the *A. thaliana* OTU2 has a relatively shorter N-terminus and does not interact with CDC48 (Figure S8 and data not shown). These results suggest potential mechanistic differentiation, such as a requirement for additional factor(s) in *A. thaliana* OTU2 recruitment by CDC48.

The human K48-specific OTUB1 plays an important role in DNA damage responses by directly inhibiting UBC13 (UBE2N) activity (Nakada et al., 2010); a non-catalytic function that utilizes K48-linkage recognition is critical for this inhibition (Juang et al., 2012; Wiener et al., 2012). Similar non-catalytic inhibition of processes mediated by the UBE2D and UBE2E families by OTUB1 is also likely (Nakada et al., 2010; Juang et al., 2012; Sun et al., 2012; Wiener et al., 2012). Because potential *A. thaliana* orthologs in the UBC13 and UBE2D-E families (http://www.arabidopsis.org/) exist, it would be interesting to examine whether a similar E2-inhibition function is associated with *A. thaliana* OTU1 and whether it is also involved in DNA damage responses. However, given its strong and moderate cleavage activities for linear and K63-linked chains, respectively, in addition to the K48 linkage catalysis and recognition, *A. thaliana* OTU1 may also be involved in other functions, such as modulating linear chain-mediated signaling.

Because only *A. thaliana* OTLD1 has been examined, the plant OTU DUBs contribute little to the functional implications of cross-species conserved OTU DUBs. However, *A. thaliana* OTLD1 (the potential *H. sapiens* OTUD5/DUBA ortholog) likely acts in a histone-modifying repressor complex that harbors histone lysine demethylase KDM1C to suppress specific gene expression through histone deubiquitylation and demethylation (Krichevsky et al., 2011). Interestingly, *A. thaliana* OTU5 (the potential *H. sapiens* OTUD6A/6B ortholog) has been observed in nuclear protein complex(es) and is involved in modulating histone marks on major flowering repressors to regulate flowering time (Radjacommare and Fu, unpublished results). These results suggest that OTU DUBs may be involved in chromatin modification-mediated transcriptional regulation in addition to their predominant roles in cellular signaling. It would be interesting to examine whether the corresponding *S. cerevisiae* and/or *H. sapiens* orthologs also have similar functions and whether these orthologs are associated with evolutionarily and functionally conserved protein complexes.

## Materials and methods

### Bioinformatics

The *A. thaliana* and *O. sativa OTU* loci were identified through reiterative searches of the TAIR (http://www.arabidopsis.org/index.jsp) and NCBI (http://blast.ncbi.nlm.nih.gov/) databases using the *H. sapiens* OTUB1 and OTUB2 and *S. cerevisiae* Otu1 sequences as the initial queries. The protein domains were identified using SMART from the ExPASy server (http://expasy.org/tools/). Routine DNA and protein sequence analyses were performed using GCG Version 11.1.3-UNIX (Accelrys Inc.).

To generate the phylogenetic tree, the OTU domain sequences were aligned using MUSCLE with default settings (Edgar, 2004). The resulting alignment was used to infer the phylogeny using maximum likelihood and Bayesian methods. We used the PhyML program for the maximum likelihood method (Guindon and Gascuel, 2003). To estimate the level of support for each internal branch, we generated 1000 non-parametric bootstrap samples of the alignment using the SEQBOOT program from the PHYLIP package (Felsenstein, 1989), and we repeated the phylogenetic inference as described above. For the Bayesian approach, we used the program MrBayes (Ronquist and Huelsenbeck, 2003; Altekar et al., 2004) to infer the posterior probability distributions. The accession numbers for the OTU-containing sequences from *O. sativa*, *H. sapiens*, and *S. cerevisiae* with abbreviated binominal name prefixes are as follows: AK120577 (Os01g0900900), EAY84610 (Os02g06890), BAD38558 (Os02g31830), BAD26139 (Os02g32180), EEC73285 (Os02g32190), BAD26147 (Os02g32280), AK067291 (Os02g0168600), NP_001046946 (Os02g0513800), AK119352 (Os02g0819500), NP_001049654 (Os03g0266000), AK072986 (Os03g0589300), AK071971 (Os03g0859800), AK240901 (Os04g32970), AK101471 (Os04g0414100), AK066247 (Os04g0619500), AK107489 (Os04g0652600), AK103099 (Os04g0670400), AK073551 (Os06g0669800), EEE69074 (Os08g42540), NP_001062186 (Os08g0506000), NP_001063527 (Os09g0487700), Q9NP73 (HsALG13), NP_060140 (HsOTUB1), NP_075601 (HsOTUB2), NP_001138845 (HsOTUD1), Q5T2D3 (HsOTUD3), EAX05048 (HsOTUD4), NP_060072 (HsOTUD5), Q7L8S5 (HsOTUD6A), AAH29760 (HsOTUD6B), NP_570971 (HsOTUD7A), NP_064590 (HsOTUD7B), Q96BN8 (HsOTULIN/FAM105B), NP_006281 (HsTNFAIP3/A20), NP_079330 (HsVCPIP1), NP_061036 (HsYOD1), CAB64449 (HsZRANB1), P43558 (ScOTU1), and P38747 (ScOTU2).

### Recombinant protein purification

To express the various His- and His/GST-tagged OTU proteins, the corresponding full-length coding regions were PCR-amplified using PfuTurbo (Agilent Technologies) and cloned in-frame into pET28 or pET42, respectively (EMD Millipore). The clones, specific primers designed to add appropriate restriction sites for cloning, template cDNA libraries, and vectors are listed (Table S1). To express the site-specific OTU variants, mutagenesis was performed using PfuTurbo in accordance with the manufacturer's instructions (Agilent Technologies); the specific primers are listed (Table S1). The sequences for the expression constructs were verified by DNA sequence analysis using an ABI PRISM 3700 DNA Analyzer (Life Technologies). Recombinant protein expression in *E. coli* BL21 (DE3) (Novagen) and purification has been previously described (Fatimababy et al., 2010).

### In vitro deubiquitylation assay

The *in vitro* deubiquitylation assay was performed as previously described (Balakirev et al., 2003). The purified recombinant OTU proteins and their variants were incubated at a concentration of 300 nM each with the different substrates in reaction buffer (150 mM NaCl, 0.5 mM DTT, and 20 mM Tris-HCl, pH 8) at 37°C for 1 h. To determine the optimal pH, the cleavage reactions were performed in buffers containing 150 mM NaCl and 0.5 mM DDT at different pH values (20 mM acetate buffer at pH 4.5, 20 mM phosphate buffer at pH 6.5, 20 mM Tris-HCl buffer for pH 7.5–8.5, and 20 mM 3-cyclohexylamino-1-propanesulfonic acid for pH 9.5–10.5). The hydrolysis assay was performed at 37°C for 20 min. The reactions were stopped by boiling the samples for 5 min in 2× SDS-PAGE sample buffer. The inputs and cleavage products were separated by SDS-PAGE and detected by immunoblotting with rabbit polyclonal antisera raised against *H. sapiens* UB (sc-9133) or mouse monoclonal antisera raised against the influenza hemagglutinin (HA) epitope (sc-7392) (Santa Cruz Biotechnology). The input protein concentrations were determined using the protein assay reagent (Bio-Rad Laboratories). The substrates were used in 250 ng quantities for the K48- and K63-tetraubiquitin chains (Boston Biochem); 100, 200, and 250 ng for the purified recombinant linear UB dimers, trimers, and tetramers, respectively; and 250 ng for the purified recombinant HA-tagged UB, RUB, and SUMO fusion proteins.

### UB chain binding

The GST pull-down assay and detection of pulled-down products by immunoblotting has been described previously (Fatimababy et al., 2010; Lin et al., 2011). The Lys48- and Lys63-linked UB chains (Ub2-7) were purchased from Boston Biochem.

### Accession numbers

The sequence data from this article can be found in the Arabidopsis Genome Initiative or GenBank/EMBL databases under the accession numbers listed in Table 1.

## Author contributions

Hongyong Fu and Ramalingam Radjacommare designed the research; Ramalingam Radjacommare and Raju Usharani performed the research; Hongyong Fu, Ramalingam Radjacommare, and Chih-Horng Kuo analyzed the data; and Hongyong Fu wrote the article.

### Conflict of interest statement

The authors declare that the research was conducted in the absence of any commercial or financial relationships that could be construed as a potential conflict of interest.

## Supplementary material

Figure S1 **Comparison of the exon and intron organizations of the *A. thaliana* and *O. sativa OTU*-containing loci in each phylogenetic clade**.

Figure S2 **Expression of various *A. thaliana OTU* loci across a wide variety of tissues was detected using GENEVESTIGATOR**.

Figure S3 **Relatively constitutive expression of various *A. thaliana OTU* loci was observed by RT-PCR analyses**.

Figure S4 **The protein sequence alignment of the OTU domains from *A. thaliana* OTU1-5, OTLD1, OTU7-12, and *H. sapiens* OTUB1**.

Figure S5 ***A. thaliana* OTU1, OTU3, and OTU4c display cleavage activities for peptide bond-linked UB polymers**.

Figure S6 ***A. thaliana* OTU3, OTU4c, OTU7a, and OTU9-10 cleave UB- and RUB- but not SUMO-GST fusion proteins**.

Figure S7 **Optimal cleavage activities of various *A. thaliana* OTU proteins for K48- and K63-linked ubiquitin tetramer were observed at neutral pH**.

Figure S8 **Potential orthologs from different species within the conserved phylogenetic clades have similar domain organizations and extended sequence similarities**.

Table S1 ***E. coli* intermediate and final constructs used to express *A. thaliana* OTU-deubiquitylation proteins and their test substrates**.
