# Supplementary material for: Distinct phylogenetic relationships and biochemical properties of Arabidopsis ovarian tumor-related deubiquitinases support their functional differentiation
Source: Front Plant Sci. 2014 Mar 12;5:84. doi: 10.3389/fpls.2014.00084 (PMC3950621; doi:10.3389/fpls.2014.00084)
Supplement: Figure S1 — Comparison of the exon and intron organizations of the A. thaliana and O. sativa OTU-containing loci in each phylogenetic clade. [file Presentation1.PDF]

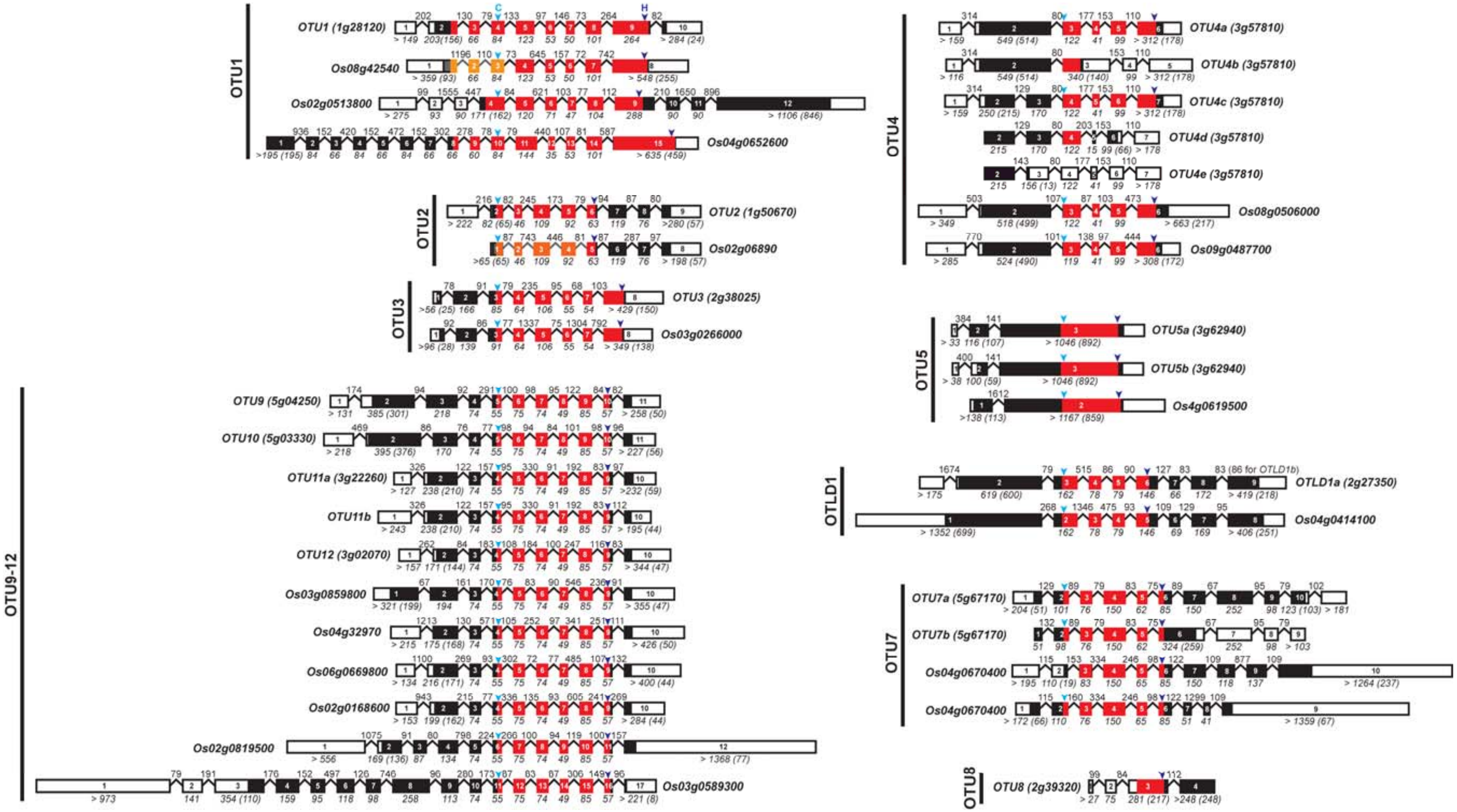

Figure S1 | Comparison of the exon and intron organizations of the *A. thaliana* and *O. sativa OTU*-containing loci in each phylogenetic clade. Exons and introns are denoted by boxes and lines, respectively, with sizes indicated in bp. The unfilled boxes are untranslated regions, and the filled boxes are coding regions; the OTU domains are colored in red. The exon numbers are marked inside the boxes. The blue and purple arrows indicate the codons for the catalytically active cysteine and histidine residues in the OTU domains, respectively. The exon and intron organizations of the *O. sativa OTU* loci were determined by comparison them with the corresponding cDNAs in the GenBank database. The coding exons are colored in grey and orange (denoted for the OTU domain) for *O. sativa OTU1* Os08g42540 and *OTU2* Os02g06890, which were putatively annotated using heterologous cDNAs because only partial rice cDNAs are available for these two loci. The accession numbers for the

genomic sequences/corresponding cDNAs used to annotate the analyzed *O. sativa* loci in parentheses are as follows: AP004863/AK062817 (Os02g06890), AP005071/AK067291 (Os02g0168600), AP004062/AK120082 (Os02g0513800), AP004878/AK119352 (Os02g0819500), AC135208/AK104949 (Os03g0266000), AC135502/AK072986 (Os03g0589300), AC092263/AK071971 (Os03g0859800), AL607005/AK240901 (Os04g32970), AL662945/AK101471 (Os04g0414100), AL662970/AK066247 (Os04g0619500), AL606690/AK107489 (Os04g0652600), AL732356/AK103099 (Os04g0670400), AP003635/AK073551 (Os06g0669800), AP004464/AK224072 (Os08g42540), AP005245/AK073328 (Os08g0506000), and AC108758/AK070222 (Os09g0487700). The heterologous cDNA accession numbers for the *O. sativa OTU1* and *OTU2* annotations are AY108350 and EZ170803 from *Zea mays* and *Artemisia annua*, respectively.

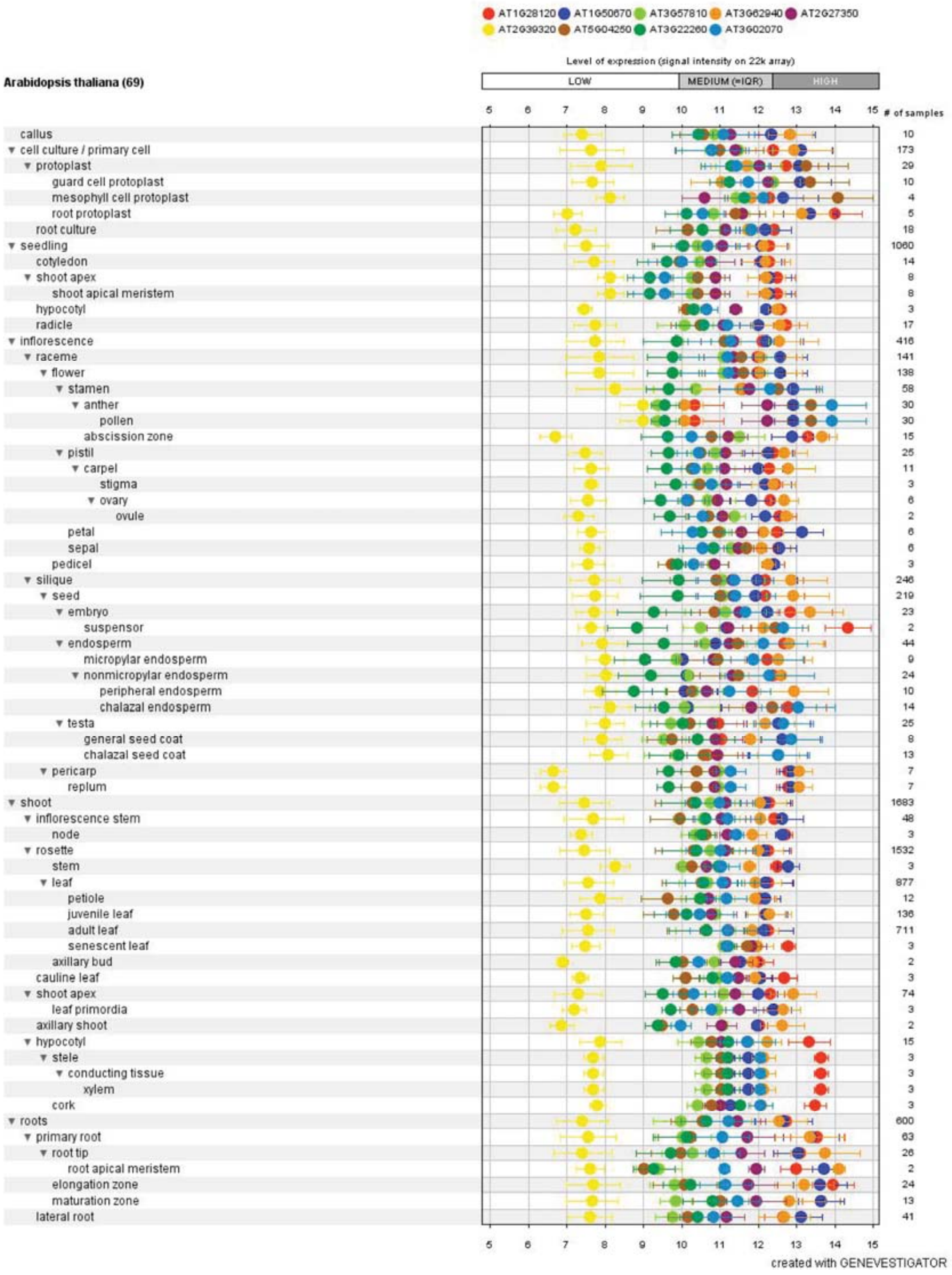

Figure S2 | Expression of various *A. thaliana OTU* loci across a wide variety of tissues was detected using GENEVESTIGATOR. The expression of various *A. thaliana OTU* loci was examined using GENEVESTIGATOR (https://www.genevestigator.com/gv/plant.jsp) and 4,399 samples with a Col-0 background in ATH1:22k array platform. Only the data for probes that target a single gene in the genome were included.

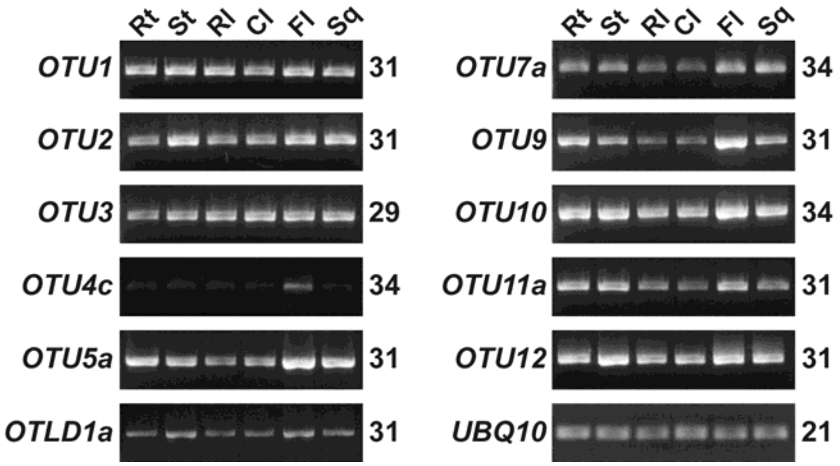

Figure S3 | Relatively constitutive expression of various *A. thaliana OTU* loci was observed by RT-PCR analyses. The expression of the 11 active *A. thaliana OTU* loci was analyzed by RT-PCR using total RNA isolated from seedling roots (Rt), stems (St), rosette leaves (Rl), cauline leaves (Cl), flower clusters (Fl), and green siliques (Sq) 35 days after stratification (DAS) from Col-0 plants grown under long-day conditions (16-h light/8-h dark) at 22°C. The corresponding primer pairs used to clone the His-tagged or GST-tagged (for *OTLD1a*) recombinant constructs were used (**Table S1**). The expression level of *UBQ10* was used as the first-strand cDNA input control. PCR cycle numbers are indicated on the right.

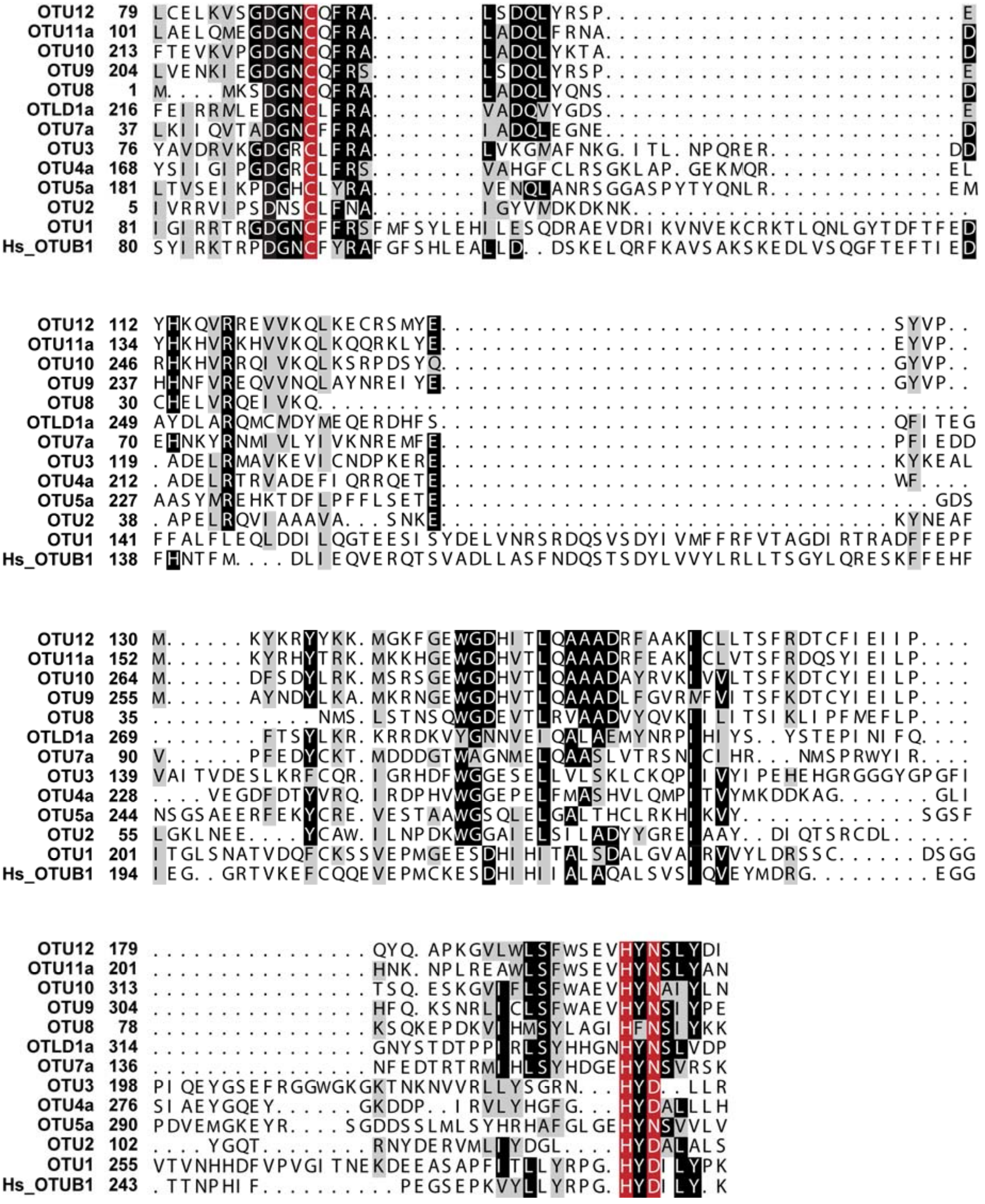

Figure S4 | The protein sequence alignment of the OTU domains from *A. thaliana* OTU1-5, OTLD1, OTU7-12, and *H. sapiens* OTUB1. The multiple sequence file, generated by GCG PileUp, is displayed using BoxShade 3.21 (http://www.ch.embnet.org/software/BOX_form.html). Identical and similar residues are shown in reverse type and shaded boxes, respectively. The gaps are identified by dots. The residues in red reverse type are the conserved cysteine, histidine, and aspartate/asparagine, that are predicted to form the catalytic triad. The coordinates of the individual sequences are indicated on the left.

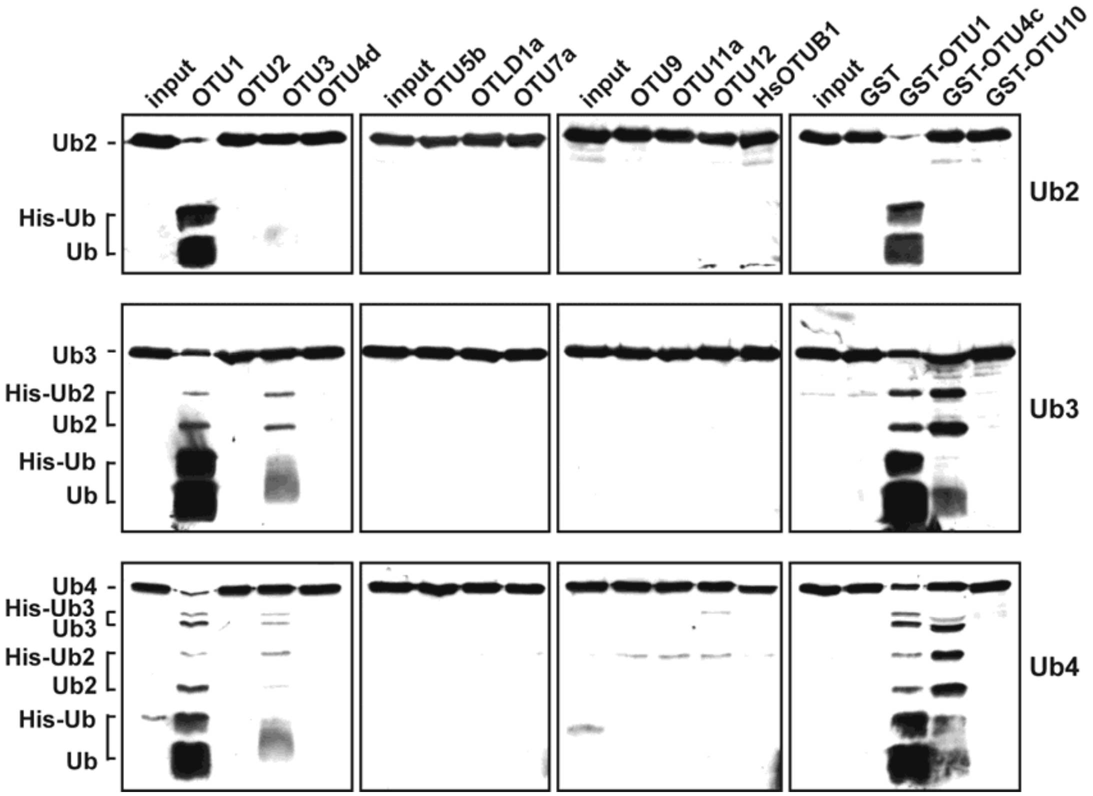

Figure S5 | *A. thaliana* OTU1, OTU3, and OTU4c display cleavage activities for peptide bond-linked UB polymers. The cleavage activities of the *A. thaliana* OTU proteins for peptide bond-linked linear UB dimers (Ub2), trimers (Ub3), and tetramers (Ub4) were analyzed using purified His- (OTU1-3, OTU4d, OTU5b, OTLD1a, OTU7a, OTU9, OTU11a, and OTU12) or His/GST-tagged (OTU1, OTU4c, and OTU10) forms. HsOTUB1 was analyzed for comparison. GST was included as a negative control for His/GST-tagged OTU proteins. A substrate incubated without enzymes was used as a negative input control (input). The inputs and their cleavage products are labeled on the left as His-tagged and free UB monomers (His-Ub and Ub), dimers (His-Ub2 and Ub2), and trimers (His-Ub3 and Ub3) and were visualized using α-UB.

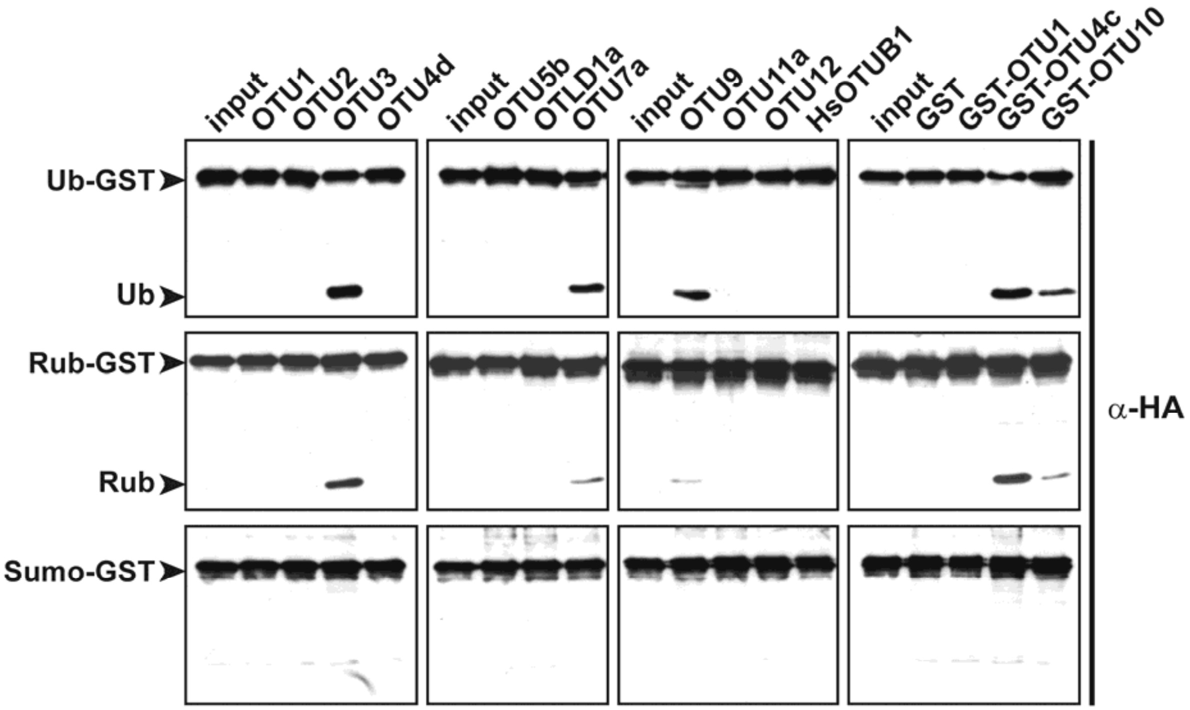

Figure S6 | *A. thaliana* OTU3, OTU4c, OTU7a, and OTU9-10 cleave UB- and RUB- but not SUMO-GST fusion proteins. The cleavage activities of the *A. thaliana* OTU proteins for HA-tagged UB-, RUB-, and SUMO-GST fusion proteins were analyzed using purified His- (OTU1-3, OTU4d, OTU5b, OTLD1a, OTU7a, OTU9, OTU11a, and OTU12) or His/GST-tagged (OTU1, OTU4c, and OTU10) forms. HsOTUB1 was analyzed for comparison. GST was included as a negative control for the His/GST-tagged OTU proteins. A substrate incubated without enzymes was used as a negative input control (input). The inputs and their cleavage products are labeled on the left as Ub and Rub and were visualized by immunoblotting with antisera against the HA tag (α-HA).

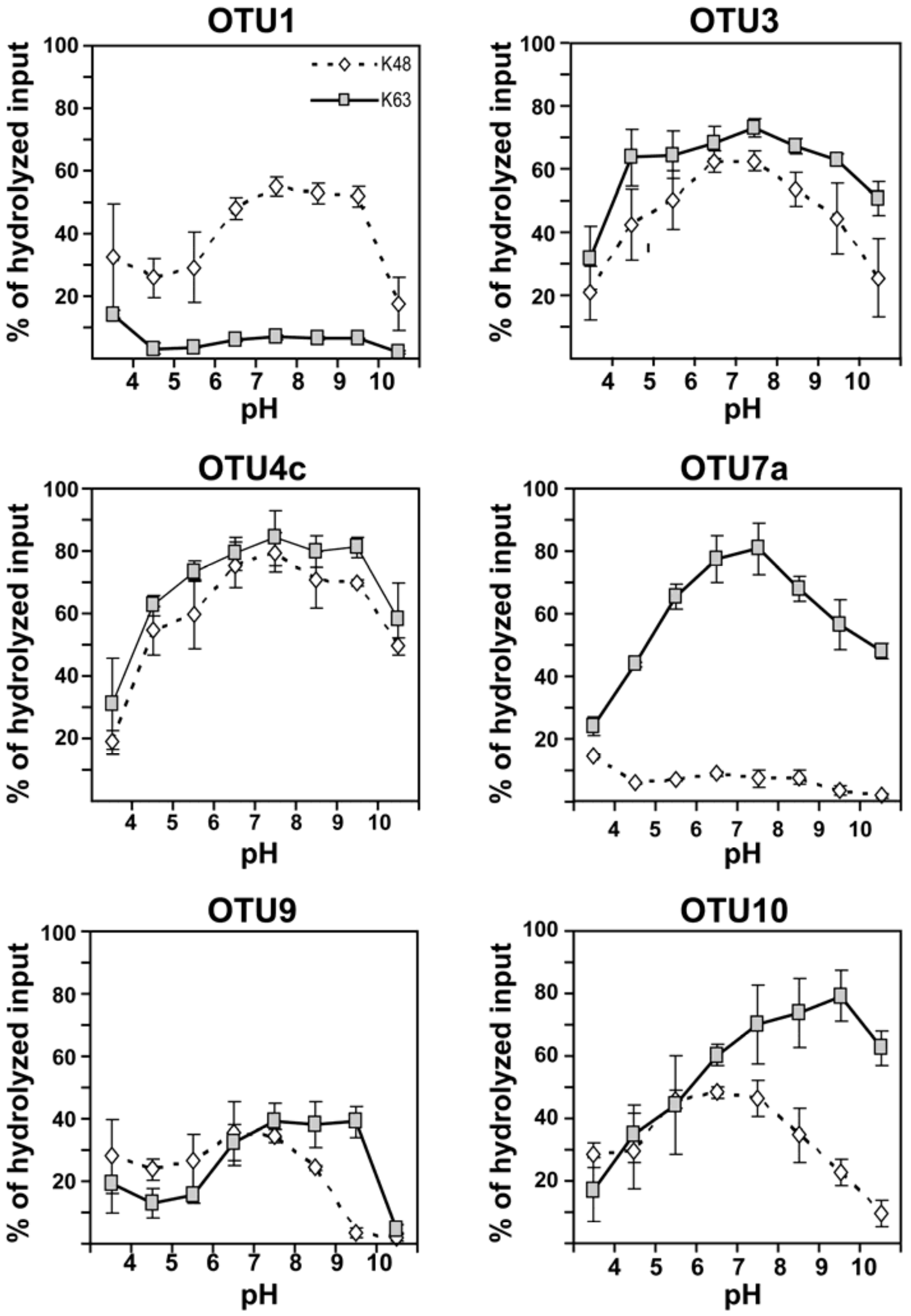

Figure S7 | Optimal cleavage activities of various *A. thaliana* OTU proteins for K48- and K63-linked ubiquitin tetramers were observed at neutral pH. The cleavage activities for K48- and K63-linked ubiquitin tetramers by purified His- (OTU1, OTU3, OTU7a, and OTU9) or GST-tagged (OTU4c and OTU10) forms were analyzed. The average cleavage activities were determined in three independent experiments by monitoring the percentage reduction of input substrates by immunoblotting with antisera against human ubiquitin. The input signal reductions were quantitated through chemiluminescence using G-Box (Syngene, Cambridge, England). The error bars represent the standard deviations.

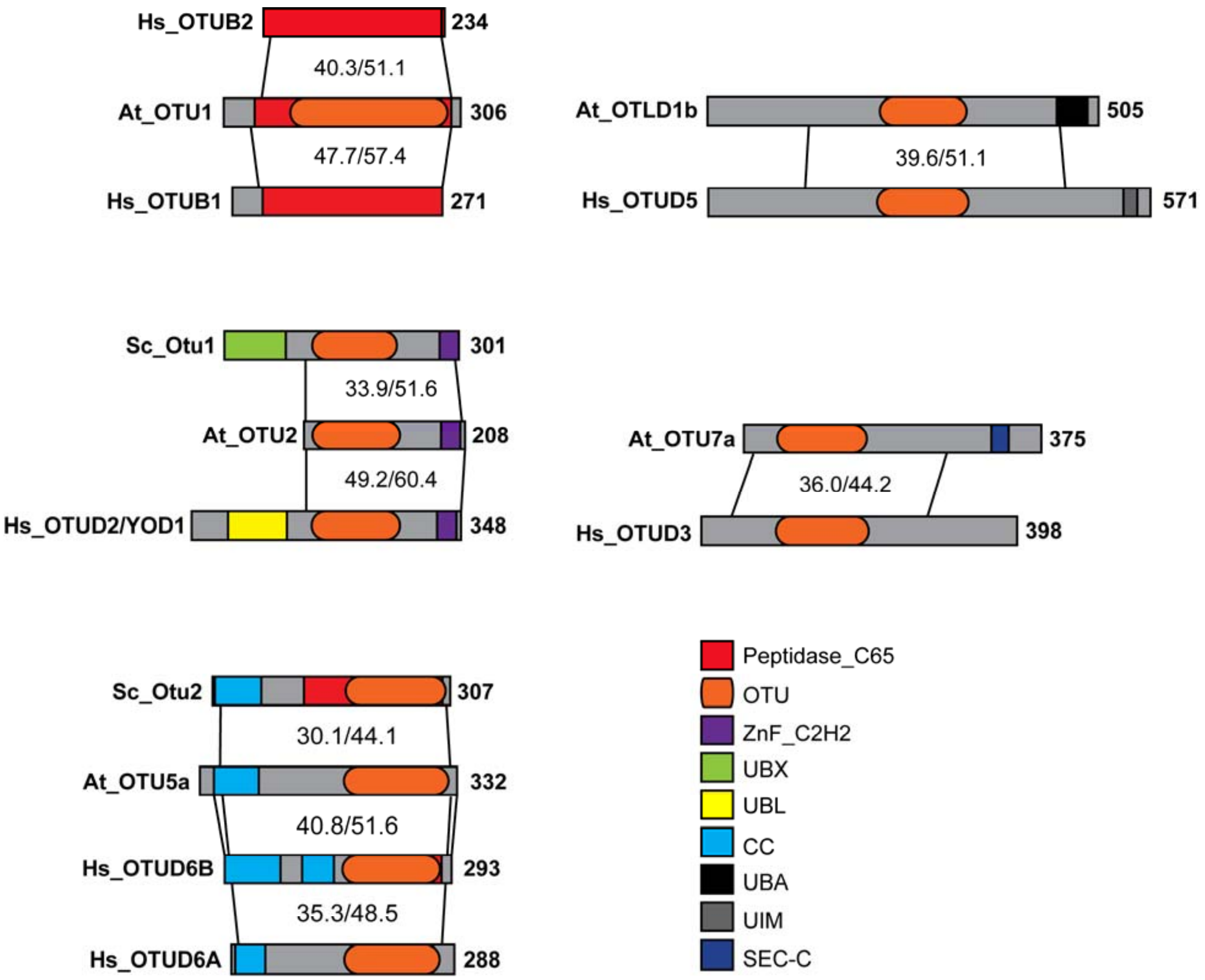

Figure S8 | Potential orthologs from different species within the conserved phylogenetic clades have similar domain organizations and extended sequence similarities. The domain organizations and percentages of peptide sequence identities/similarities were compared for potential OTU orthologs from *A. thaliana*, *S. cerevisiae*, and *H. sapiens* in five conserved phylogenetic clades. The protein domains were identified using SMART from the ExPASy server (http://expasy.org/tools/). The color-coded domains are indicated on the lower right. Domain accessions: Peptidase_C65 (PF10275), OTU (PF02338), ZnF_C2H2 (SM000355), UBL, SM000213, CC, coiled-coil, UBA, (SCOP dlifya), and SEC-C (PF02810). The UIM of OTUD5 was described by Kayagaki et. al., 2007 (Science 318, 1628-1632). The UBX of *S. cerevisiae* Otu1 was described by Rumpf and Jentsch, 2006 (Mol. Cell 21, 261-269). The percentages of peptide sequence identities/similarities indicated were determined by comparison with the *A. thaliana* sequences using the BESTFIT program in GCG Version 11.1.3-UNIX. The left and right lines connecting the *S. cerevisiae* or *H. sapiens* sequences to *A. thaliana* sequences delimit the pairwise BESTFIT sequence borders.

Table S1 | *E. coli* intermediate and final constructs used to express *A. thaliana* OTU-deubiquitylation proteins and their test substrates.

Name	Locus or accession/template[a]	Vector, sites[b]	PCR primers for amplification or mutagenesis[c]
OTU1	At2g28120/CD4-14	pET28a, B/X	5’ TAG**gatcc**ATGCAGAATCAGATTGATATGGTG 3’<br>5’ TAT**c**TC**g**A**g**TGATCATTTCCCCACATTGTCTGATACC 3’
OTU1-D89E	At2g28120/OTU1	pET28a, B/X	5’ GGCGAACAAGAGGAGA**a**GGAAATTGCTTCTTCC 3’<br>5’ GGAAGAAGCAATTTCC**t**TCTCCTCTTGTTCGCC 3’
OTU1-C92S	At2g28120/OTU1	pET28a, B/X	5’ CAAGAGGAGATGGAAAT**a**GCTTCTTCCGAAGTTTTATG 3’<br>5’ CATAAAACTTCGGAAGAAGC**t**ATTTCCATCTCCTCTTG 3’
OTU1-H288R	At2g28120/OTU1	pET28a, B/X	5’ CTGTATCGTCCAGGCC**g**TTACGATATCCTCTAC 3’<br>5’ GTAGAGGATATCGTAA**c**GGCCTGGACGATACAG 3’
GST-OTU1	At2g28120/OTU1	pET42a, B/X	Mobilized from OTU1
OTU2	At1g50670/CD4-12	pET28a, B/X	5’ TAT**ggatc**CATGGAAGGAATCATTGTGAGAAGAG 3’<br>5’ CATC**tcgag**CGTGGTTTACTTTCTTGTTTCTTGGAACTG 3’
GST-OTU2	At1g50670/OTU2	pET42a, B/X	Mobilized from OTU2
GST-OTU2-C16S	At1g50670/ GST-OTU2	pET42a, B/X	5’ CCATCTGATAACAGT**a**GTCTCTTCAATGCAATCGG 3’<br>5’ CCGATTGCATTGAAGAGAC**t**ACTGTTATCAGATGG 3’
GST-OTU2-C63S	At1g50670/ GST-OTU2	pET42a, B/X	5’ GGAAGCTCAATGAAGAATAT**a**GTGCTTGGATTCTCAATCCAG 3’<br>5’ CTGGATTGAGAATCCAAGCAC**t**ATATTCTTCATTGAGCTTCC 3’
GST-OTU2-C99S	At1g50670/ GST-OTU2	pET42a, B/X	5’ GACATTCAAACTAGTCGA**a**GTGACTTGTATGGACAGAC 3’<br>5’GTCTGTCCATACAAGTCAC**t**TCGACTAGTTTGAATGTC 3’
GST-OTU2-C179S	At1g50670/ GST-OTU2	pET42a, B/X	5’ CAAACTTCACTCTACGT**a**GCGGTGTTTGCCAAATTGG 3’<br>5’ CCAATTTGGCAAACACCGC**t**ACGTAGAGTGAAGTTTG 3’
GST-OTU2-C182S	At1g50670/ GST-OTU2	pET42a, B/X	5’ CTCTACGTTGCGGTGTT**a**GCCAAATTGGAGTTATTGG 3’<br>5’ CCAATAACTCCAATTTGGC**t**AACACCGCAACGTAGAG 3’
GST-OTU2-H195R	At1g50670/ GST-OTU2	pET42a, B/X	5’ CAAAAGGAAGCTGTGGAAC**g**TGCTCAAGCAACTGGTC 3’<br>5’ GACCAGTTGCTTGAGCA**c**GTTCCACAGCTTCCTTTTG 3’
GST-OTU2-H201R	At1g50670/ GST-OTU2	pET42a, B/X	5’ CATGCTCAAGCAACTGGTC**g**TGTTAATTTTCAAGAATAC 3’<br>5’ GTATTCTTGAAAATTAACA**c**GACCAGTTGCTTGAGCATG 3’
GST-OTU2-C63S-C99S	At1g50670/ GST-OTU2-C63S	pET42a, B/X	5’ GACATTCAAACTAGTCGA**a**GTGACTTGTATGGACAGAC 3’<br>5’GTCTGTCCATACAAGTCAC**t**TCGACTAGTTTGAATGTC 3’
GST-OTU2-C179S-H195R	At1g50670/ GST-OTU2-C179	pET42a, B/X	5’ CAAAAGGAAGCTGTGGAAC**g**TGCTCAAGCAACTGGTC 3’<br>5’ GACCAGTTGCTTGAGCA**c**GTTCCACAGCTTCCTTTTG 3’
GST-OTU2-CΔ1	At1g50670/ GST-OTU2	pET42a, B/X	5’ TAT**ggatc**CATGGAAGGAATCATTGTGAGAAGAG 3’<br>5’ TTG**ctcg**A**g**ACC**t**CAACGTAGAGTGAAGTTTGCAGTATC 3’
GST-OTU2-CΔ2	At1g50670/ GST-OTU2	pET42a, B/X	5’ TAT**ggatc**CATGGAAGGAATCATTGTGAGAAGAG 3’<br>5’ CAT**c**TC**g**A**g**ATCTT**a**CTCGGCCCCTTCAAATGGAGACAG 3’

GST-OTU2-NΔ1	At1g50670/	pET42a, B/X	5’AGTgGATccGACTTGTATGGACAGACGAG 3’
	GST-OTU2		5’ CATCtcgagCGTGGTTTACTTTCTTGTTTCTTGGAACTG 3’
OTU3	At2g38025/CD4-12	pET28a, B/X	5’ TCCggatCcATGGAGCTCAAATCTTCTTCCAACAA 3’
			5’ TTCctcGAgCATATCAATAAATGTCAACGGCAGAGG 3’
GST-OTU3	At2g38025/OTU3	pET42a, B/X	Mobilized from OTU3
OTU4a	At3g57810/CD4-14	pET28a, R/X	5’ ATAgAAttcATGATGATTTGTTACTCTCCAATTAC 3’
			5’ AAACtcgAgCATCATCAACACCTGACAAAAGTCAA 3’
OTU4c	At3g57810/CD4-14	pET28a, R/X	5’ ATAgAAttcATGATGATTTGTTACTCTCCAATTAC 3’
			5’ AAACtcgAgCATCATCAACACCTGACAAAAGTCAA 3’
OTU4d	At3g57810/CD4-14	pET28a, R/X	5’ ATAgAAttcATGATGATTTGTTACTCTCCAATTAC 3’
			5’ AAACtcgAgCATCATCAACACCTGACAAAAGTCAA 3’
GST-OTU4c	At3g57810/	pET42a, R/X	Mobilized from OTU4c
	OTU4c		
GST-OTU4c-NΔ	At3g57810/	pET42a, R/X	5’ AGTgaaTTcTCAGGACTTTCAAGAAGACATTATACC 3’
	GST-OTU4-iia		5’ AAACtcgAgCATCATCAACACCTGACAAAAGTCAA 3’
GST-OTU4c-C136S	At3g57810/	pET42a, R/X	5’ CCGGGGGATGGCAGAaGTTTGTTTCGTTCTGTAG 3’
	GST-OTU4c		5’ CTACAGAACGAAACAAACtTCTGCCATCCCCCGG 3’
OTU5a	At3g62940/CD4-14	pET28a, R/S	5’ CTTgAaTTcATGGCAGACAATAAAACATCTGAAGG 3’
			5’ GATgAgcTCCATCATCCATCATCCGGTGATGTTGTT 3’
OTU5b	At3g62940/CD4-14	pET28a, R/S	5’ GAAGAAttcCCCATGGATGCTTCTCAGGAGCAACAACA 3’
			5’ GATgAgcTCCATCATCCATCATCCGGTGATGTTGTT 3’
GST-OTU5a	At3g62940/OTU5a	pET42a, R/S	Mobilized from OTU5a
GST-OTU5b	At3g62940/	pET42a, R/S	Mobilized from OTU5b
	OTU5b		
OTLD1a	At2g27350/	pET28a, R/S	Mobilized from GST- OTLD1a
	GST- OTLD1a		
OTLD1a-CΔ1	At2g27350/	pET28a, R/S	5’ AGAgaattCATGACTCGGATTTTGGTTCAAAGAGG 3’
	GST- OTLD1a		5’ TTCcTcgAgTGCCTaTGCTGGTTTCGAATCGCTGCC 3’
GST- OTLD1a	At2g27350/	pET42a, R/S	5’ AGAgaattCATGACTCGGATTTTGGTTCAAAGAGG 3’
	CD4-14		5’ AATctcGAGCAATTCATGTTACATTCTCTATTC 3’
GST- OTLD1a-NΔ1	At2g27350/	pET42a, R/S	5’ TGTGAattcCCAGTTAGCGGTGGCAGTAACTCTG 3’
	GST- OTLD1a		5’ AATctcGAGCAATTCATGTTACATTCTCTATTC 3’
GST- OTLD1a-NΔ2	At2g27350/	pET42a, R/S	5’ GCAGAatTcAGATATTCAAAAGGATTTGAAATTAGG 3’
	GST- OTLD1a		5’ AATctcGAGCAATTCATGTTACATTCTCTATTC 3’
GST- OTLD1a-CΔ1	At2g27350/	pET42a, R/S	Mobilized from OTLD1a-CΔ1
	GST- OTLD1a		
GST- OTLD1a-CΔ2	At2g27350/	pET42a, R/S	5’ AGAgaattCATGACTCGGATTTTGGTTCAAAGAGG 3’
	GST- OTLD1a		5’ TGGCTcgagCCATTaCATAAGATACTCAGCACGAGAAGC 3’

GST- OTLD1a-CΔ3	At2g27350/	pET42a, R/S	5’ AGAgaattCATGACTCGGATTTTGGTTCAAAGAGG 3’
	GSTa- OTLD1a		5’ AAActCgAGTCaTGCACCAACTGTCAACCGATGTGG 3’
GST- OTLD1a-CΔ4	At2g27350/	pET42a, R/S	5’ AGAgaattCATGACTCGGATTTTGGTTCAAAGAGG 3’
	GST- OTLD1a		5’ AAActcgagATTTCaATCTTCCAACATCCGCCTAATTTC 3’
GST-	At2g27350/	pET42a, R/S	5’ GCAGAatTcAGATATTCAAAAGGATTTGAAATTAGG 3’
OTLD1a-OTUf	GST- OTLD1a		5’ TTCcTcgAgTGCCTaTGCTGGTTTCGAATCGCTGCC 3’
OTU7a	At5g67170/CD4-14	pET28a, B/X	5’ GATggatccATGGCGAAAACTAAACAACAGAAATCTAGG 3’
			5’ ATACTcgAgTTATGTCTTCGCCTTGAGTGAGTGAGAC 3’
OTU7a-C48S	At5g67170/OTU7a	pET28a, B/X	5’ GTGACTGCTGACGGTAATaGTTTCTTCAGAGCAATTGCTG 3’
			5’ CAGCAATTGCTCTGAAGAAACtATTACCGTCAGCAGTCAC 3’
GST-OTU7a	At5g67170/OTU7a	pET42a, B/X	Mobilized from OTU7a
OTU9	At5g04250/CD4-14	pET28a, R/S	5’ GAATtcATGGGGTATGAGCCTGATCCAGATGCTC 3’
			5’ CCTCCATCAGCAGAAAGAGAGCTCAG 3’
OTU9-NΔ1-100	At5g04250/OTU9	pET28a, R/S	5’ CCTgAattcGGACAGGAAAATCAAGGGGAAGCC 3’
			5’ CCTCCATCAGCAGAAAGAGAGCTCAG 3’
OTU9-NΔ1-172	At5g04250/OTU9	pET28a, R/S	5’ ATAgaatTcGCTCATGTGCCGAAAATCAATGGGG 3’
			5’ CCTCCATCAGCAGAAAGAGAGCTCAG 3’
GST-OTU9	At5g04250/OTU9	pET42a, R/S	Mobilized from OTU9
OTU10	At5g03330/CD4-14	pET28a, R/H	5’ TGTgaattcATGGTGTCACATGAAGAGAACACAAG 3’
			5’ ATGAagCttTGGTCTGTCTAGTTTCCGAAACGCCACC 3’
GST-OTU10	At5g03330/OTU10	pET42a, R/H	Mobilized from OTU10
GST-OTU10-NΔ1-1	At5g03330/	pET42a, R/H	5’ TATgaaTtcGAATGGATAGGAAATGATAATGATC 3’
25	GST-OTU10		5’ ATGAagCttTGGTCTGTCTAGTTTCCGAAACGCCACC 3’
GST-OTU10-NΔ1-1	At5g03330/	pET42a, R/H	5’ TTTGaAttcAGATTGAATCAAATGGTTCCCATTCC 3’
73	GST-OTU10		5’ ATGAagCttTGGTCTGTCTAGTTTCCGAAACGCCACC 3’
OTU11a	At3g22260/CD4-14	pET28a, B/Sal	5’ CCAggatCcATGGATGAAAACCATAGGAATCCATTTG 3’
			5’ ACTgTcgAcCCAATACTAGAAGAGCCAATGCTTCC 3’
GST-OTU11a	At3g22260/	pET42a, B/Sal	Mobilized from OTU11a
	OTU11a		
OTU12	At3g02070/CD4-14	pET28a, R/H	5’ CTTgaATTcATGGGAGACTCTTCGAGTTCAACCTC 3’
			5’ GCGaagCTTCTAGAACAACCAATGTTTTCTCTTTGG 3’
GST-OTU12	At3g02070/OTU12	pET42a, R/H	Mobilized from OTU12
HsOTUB1	AK000120/	pET28a, B/X	5’ TGTggAtccATGGCGGCGGAGGAACCTCAGCAG 3’
	1st strand cDNAs		5’GGGCTcGAGCCAGCCCTATTTGTAGAGGATATC 3’
UB2	JX089332/CD4-22	pET28a, B/H	5’ GTGggatccATGCAGATCTTTGTTAAGACTCTCACC 3’
			5’ GATaAgcTTGAAGAAGTTCGACTTGTCATTAGAAAG 3’
UB3	JX089333/CD4-22	pET28a, B/H	5’ GTGggatccATGCAGATCTTTGTTAAGACTCTCACC 3’
			5’ GATaAgcTTGAAGAAGTTCGACTTGTCATTAGAAAG 3’

UB4	JX089331/CD4-22	pET28a, B/H	5’ GTG**ggatcc**ATGCAGATCTTTGTTAAGACTCTCACC 3’<br>5’ GAT**a**A**gc**TTGAAGAAGTTCGACTTGTCATTAGAAAG 3’
RAD23d-1	At5g38470/CD4-14	pET28a, B/X	5’ CCC**gg**A**t**C**c**ATGAAGATTTTCGTGAAGACTCTCAG 3’<br>5’ TTGCTC**gag**TTATTGATCTTCAAACTCATGCATGTG 3’
RAD23d-2	At5g38470/ RAD23d-1	pET30a, H/X	5’ CGC**aagctt**ATGAAGATTTTCGTGAAGACTCTCAG 3’<br>5’ GTGCTC**gag**TTATTGATCCTCAAACTCATGCATGTG 3’
GST	/pET42a	pET30a, H/X	5’ GATA**ag**C**t**TATGTCCCCTATACTAGGTTATTG 3’<br>5’ AACC**t**C**g**AGTT**c**AACCATCCGATTTTGGAGGATGG 3’
HA3-UBQ	1538658/UB2	p1239, Nd/H	5’ GGA**cat**ATGCAGATCTTTGTTAAGACTCTC 3’<br>5’ GAA**a**A**g**CT**tg**A**a**ACCTCCACGCAGACGCAACAC 3’
HA3-UBQ-GST	1538658/HA3-RUB-GST	HA3-RUB-GST, Nc/H	The *Nco*I-*Hind* III fragment containing the HA3-tagged RUB fragment in HA3-RUB-GST was replaced by the HA3-UBQ fragment from HA3-UBQ
RUB	At1g31340/ CD4-13	pET28a, B/N	5’ GGTGG**at**C**c**ATGATTAAGGTGAAGACTCTCACAGG 3’<br>TCAG**gcggccg**C**tc**AACCACCCCTAAGGGCAAGAACCAGATG 3’
HA3-RUB-1	At1g31340/RUB	p1239, Nd/H	5’ GGG**ca**TATGATTAAGGTGAAGACTCTCACAGG 3’<br>5’ TAC**aagctt**GAGAAGACCAAAACCACCCCTAAGGGCAAGAACCAGATG 3’
HA3-RUB-2	At1g31340/ HA3-RUB-1	RAD23d-2, Nc/H	The *Nco*I-*Hind* III fragment containing the HA3-tagged RUB from HA3-RUB-1 was mobilized into RAD23d-2
HA3-RUB-GST	At1g31340/ HA3-RUB2	HA3-RUB-2, H/X	The *Hind*III-*Xho*I fragment containing RAD23d in HA3-RUB-2 was replaced by the similarly restricted fragment from GST
HA3-SUMO-1	At4g26840/CD4-22	p1239, Nd/H	5’ TTC**cat**ATGTCTGCAAACCAGGAGGAAGACAAG 3’<br>5’ GAA**aa**G**ctt**GGCCGTAGCACCACCACCGCTGCCACC 3’
HA3-SUMO-2	At4g26840/ HA3-SUMO-1	RAD23d-2, Nc/H	The *Nco*I-*Hind* III fragment containing the HA3-tagged SUMO from HA3-SUMO-1 was mobilized into RAD23d-2
HA3-SUMO-GST	At4g26840/ HA3-SUMO-2	HA3-SUMO-2, H/X	The *Hind*III-*Xho*I fragment containing RAD23d in HA3-SUMO-2 was replaced by the similarly restricted fragment from GST

[a]The human HsOTUB1 coding sequence was PCR-amplified using first-strand cDNAs of from human HeLa S3 cells (Stratagene).

[b]The abbreviations for restriction sites are as follows: B, *Bam*HI; N, *Not*I; Nc, *Nco*I; Nd, *Nde*I; H, *Hind*III; R, *Eco*RI; S, *Sac*I; Sal, *Sal*I; and X, *Xho*I. The sub-cloning vector p1239 (Liu et al., 1988 Curr. Biol. 8, 1300-1309) was employed as an intermediate step to add the coding sequence for the HA3 tag to ubiquitin, RUB, and SUMO1.

[c]The primer pair sequences used for PCR amplification or site-directed mutagenesis are listed. The bases in upper case and plain text are identical to their templates, and the bases in lower case and bold-face are mutated relative to their templates. The underlined bases are native or engineered restriction sites. The boxes in the 5’ primer sequences are the ATG start codons. The boxes in the 3’ primer sequences are native or engineered (indicated with lower case/bold letters) stop codons. The boxes in the yellow shaded cells are mutated native stop codons for in-frame fusion to the HA tag.
